# Maternal age is related to offspring DNA methylation: A meta‐analysis of results from the PACE consortium

**DOI:** 10.1111/acel.14194

**Published:** 2024-05-29

**Authors:** Edwina Yeung, Richard J. Biedrzycki, Laura C. Gómez Herrera, Prachand Issarapu, John Dou, Irene Fontes Marques, Sohail Rafik Mansuri, Christian Magnus Page, Justin Harbs, Dennis Khodasevich, Eric Poisel, Zhongzheng Niu, Catherine Allard, Emma Casey, Fernanda Morales Berstein, Giulia Mancano, Hannah R. Elliott, Rebecca Richmond, Yiyan He, Justiina Ronkainen, Sylvain Sebert, Erin M. Bell, Gemma Sharp, Sunni L. Mumford, Enrique F. Schisterman, Giriraj R. Chandak, Caroline H. D. Fall, Sirazul A. Sahariah, Matt J. Silver, Andrew M. Prentice, Luigi Bouchard, Magnus Domellof, Christina West, Nina Holland, Andres Cardenas, Brenda Eskenazi, Lea Zillich, Stephanie H. Witt, Tabea Send, Carrie Breton, Kelly M. Bakulski, M. Daniele Fallin, Rebecca J. Schmidt, Dan J. Stein, Heather J. Zar, Vincent W. V. Jaddoe, John Wright, Regina Grazuleviciene, Kristine Bjerve Gutzkow, Jordi Sunyer, Anke Huels, Martine Vrijheid, Sophia Harlid, Stephanie London, Marie‐France Hivert, Janine Felix, Mariona Bustamante, Weihua Guan

**Affiliations:** ^1^ Epidemiology Branch, Division of Population Health Research, Division of Intramural Research Eunice Kennedy Shriver National Institute of Child Health and Human Development Bethesda Maryland USA; ^2^ Division of Intramural Research Glotech Inc., Eunice Kennedy Shriver National Institute of Child Health and Human Development, National Institutes of Health Bethesda Maryland USA; ^3^ ISGlobal, Institute for Global Health Barcelona Spain; ^4^ Universitat Pompeu Fabra (UPF) Barcelona Spain; ^5^ CIBER Epidemiología y Salud Pública (CIBERESP) Madrid Spain; ^6^ MRC Unit the Gambia at the London School of Hygiene and Tropical Medicine (LSHTM) Banjul The Gambia; ^7^ Department of Epidemiology, School of Public Health University of Michigan Ann Arbor Michigan USA; ^8^ Generation R Study Group, Erasmus MC University Medical Center Rotterdam Rotterdam The Netherlands; ^9^ Department of Pediatrics, Erasmus MC University Medical Center Rotterdam Rotterdam The Netherlands; ^10^ Genomic Research on Complex Diseases (GRC‐Group) CSIR‐Centre for Cellular and Molecular Biology Hyderabad Telangana India; ^11^ Oslo Centre for Biostatistics and Epidemiology Oslo University Hospital Oslo Norway; ^12^ Department of Diagnostics and Intervention, Oncology Umeå University Umeå Sweden; ^13^ Environmental Health Sciences, Berkeley Public Health CERCH, University of California Berkeley California USA; ^14^ Department of Genetic Epidemiology in Psychiatry Central Institute of Mental Health, Medical Faculty Mannheim, University of Heidelberg Mannheim Germany; ^15^ Department of Population and Public Health Science, Keck School of Medicine University of Southern California Los Angeles California USA; ^16^ Centre de Recherche du Centre Hospitalier Universitaire de Sherbrooke (CHUS) Sherbrooke Quebec Canada; ^17^ Department of Epidemiology, Rollins School of Public Health Emory University Atlanta Georgia USA; ^18^ Medical Research Council Integrative Epidemiology Unit University of Bristol Bristol UK; ^19^ Bristol Medical School Population Health Sciences University of Bristol Bristol UK; ^20^ Research Unit of Population Health University of Oulu Oulu Finland; ^21^ Department of Environmental Health Sciences and Epidemiology and Biostatistics University at Albany School of Public Health Albany New York USA; ^22^ Department of Psychology University of Exeter Exeter UK; ^23^ Department of Biostatistics, Epidemiology and Informatics and Department of Obstetrics and Gynecology, Perelman School of Medicine University of Pennsylvania Philadelphia Pennsylvania USA; ^24^ MRC Lifecourse Epidemiology Centre University of Southampton Southampton UK UK; ^25^ Centre for the Study of Social Change Mumbai India; ^26^ Department of Biochemistry and Functional Genomics Centre intégré Universitaire de santé et de Services Sociaux (CIUSSS) du Saguenay‐Lac‐St‐Jean, Université de Sherbrooke Sherbrooke Quebec Canada; ^27^ Department of Laboratory Medicine CIUSSS du Saguenay‐Lac‐Saint‐Jean – Hôpital de Chicoutimi Chicoutimi Quebec Canada; ^28^ Department of Clinical Sciences, Pediatrics Umeå University Umeå Sweden; ^29^ Department of Epidemiology and Population Health Stanford University Stanford California USA; ^30^ Department of Psychiatry and Psychotherapy Central Institute of Mental Health, Medical Faculty Mannheim, University of Heidelberg Mannheim Germany; ^31^ Dean's Office, Rollins School of Public Health Emory University Atlanta Georgia USA; ^32^ Department of Public Health Sciences and the M.I.N.D. Institute, School of Medicine University of California Davis California USA; ^33^ Neuroscience Institute, University of Cape Town Cape Town South Africa; ^34^ Department of Psychiatry and Mental Health University of Cape Town Cape Town South Africa; ^35^ South African Medical Research Council (SAMRC) Unit on Risk and Resilience in Mental Disorders University of Cape Town Cape Town South Africa; ^36^ Department of Paediatrics and Child Health, Red Cross War Memorial Children's Hospital University of Cape Town Cape Town South Africa; ^37^ Bradford Institute for Health Research, Temple Bank House, Bradford Royal Infirmary Bradford UK; ^38^ Department of Environmental Sciences Vytautas Magnus University Kaunas Lithuania; ^39^ Division of Climate and Environmental Health Norwegian Institute of Public Health Oslo Norway; ^40^ IMIM‐Parc Salut Mar Barcelona Spain; ^41^ Gangarosa Department of Environmental Health, Rollins School of Public Health Emory University Atlanta Georgia USA; ^42^ Epidemiology Branch National Institute of Environmental Health Sciences, National Institutes of Health, Research Triangle Park Durham North Carolina USA; ^43^ Division of Chronic Disease Research across the Lifecourse (CoRAL); Department of Population Medicine, Harvard Medical School Harvard Pilgrim Health Care Institute Boston Massachusetts USA; ^44^ Diabetes Unit, Massachusetts General Hospital Boston Massachusetts USA; ^45^ Division of Biostatistics, School of Public Health University of Minnesota Minneapolis Minnesota USA

**Keywords:** aging, child, DNA methylation, melatonin, receptor

## Abstract

Worldwide trends to delay childbearing have increased parental ages at birth. Older parental age may harm offspring health, but mechanisms remain unclear. Alterations in offspring DNA methylation (DNAm) patterns could play a role as aging has been associated with methylation changes in gametes of older individuals. We meta‐analyzed epigenome‐wide associations of parental age with offspring blood DNAm of over 9500 newborns and 2000 children (5–10 years old) from the Pregnancy and Childhood Epigenetics consortium. In newborns, we identified 33 CpG sites in 13 loci with DNAm associated with maternal age (P_FDR_ < 0.05). Eight of these CpGs were located near/in the *MTNR1B* gene, coding for a melatonin receptor. Regional analysis identified them together as a differentially methylated region consisting of 9 CpGs in/near *MTNR1B*, at which higher DNAm was associated with greater maternal age (P_FDR_ = 6.92 × 10^−8^) in newborns. In childhood blood samples, these differences in blood DNAm of *MTNR1B* CpGs were nominally significant (*p* < 0.05) and retained the same positive direction, suggesting persistence of associations. Maternal age was also positively associated with higher DNA methylation at three CpGs in *RTEL1‐TNFRSF6B* at birth (P_FDR_ < 0.05) and nominally in childhood (*p* < 0.0001). Of the remaining 10 CpGs also persistent in childhood, methylation at cg26709300 in *YPEL3/BOLA2B* in external data was associated with expression of *ITGAL*, an immune regulator. While further study is needed to establish causality, particularly due to the small effect sizes observed, our results potentially support offspring DNAm as a mechanism underlying associations of maternal age with child health.

## Graphical abstract

The study meta‐analyzed epigenome‐wide associations of parental age with offspring blood DNAm of newborns and children (5–10 years old) from cohorts in the Pregnancy and Childhood Epigenetics consortium. DNA was derived from newborn or childhood blood samples and analyzed using the 450K or EPIC microarrays. DNAm levels were associated with maternal age but not paternal age and potentially support offspring DNAm as a mechanism underlying associations of maternal age with child health.

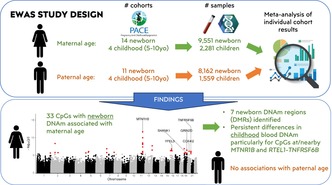

## 1 INTRODUCTION

Maternal and paternal age have steadily risen in the past few decades in many countries (Doan et al., 2022; Schmidt et al., 2012). For instance, in the United States, the median maternal age at delivery rose from 27 years in 1990 to 30 years in 2019 (Morse, 2022; Osterman et al., 2023), with similar trends in paternal age (Khandwala et al., 2017). Delays in childbearing have been attributed to the pursuit of higher education, career opportunities, housing affordability, and more stable relationships (Mills et al., 2011; Schmidt et al., 2012). Although multiple governments have enacted incentive policies in attempts to reverse these trends (Doan et al., 2022; Mills et al., 2011), there is little evidence that these trends will change.

Epidemiological studies have observed associations for both older maternal and paternal age with various adverse health outcomes in the offspring including cognitive development (Wang et al., 2022; Wu et al., 2017), cardiovascular development (Cooke & Davidge, 2019), allergies (Lu et al., 2020), and cancer (Hemminki et al., 1999), among others (Zhang et al., 2022). The mechanisms for these associations remain unclear. De novo mutations in gametes occur more frequently with advancing paternal and maternal age than with accelerated frequency for women in older age compared to men (Wong et al., 2016). Epigenetic changes may be another route through which effects occur and have been identified as a greater contributor to the aging process than genetics alone (Ge et al., 2015). Indeed, multiple mechanisms may produce aging‐related epigenetic changes in oocytes (Ge et al., 2015) and in sperm (Ashapkin et al., 2023; Oluwayiose et al., 2021). Intergenerationally, oocyte aging has been associated with differences in embryonic and placental DNA methylation (Qin et al., 2023) while, for instance, the DNA methylation related to sperm aging in mice was associated with differences of brain DNA methylation of offspring who exhibited behavioral differences (Milekic et al., 2015). Given that the embryo undergoes rapid demethylation and re‐methylation, the epigenetic alterations to offspring may also be a downstream result of aging‐reduced gene expression of proteins crucial to these early embryonic processes (Castillo‐Fernandez et al., 2020).

Despite the evidence for parental age associations with offspring health and the likely involvement of epigenetics, few studies have investigated parental age‐associated differential DNA methylation in offspring (Adkins et al., 2011; Hua et al., 2022; Markunas et al., 2016). Two of these studies used microarray technology (the Illumina 27 K and 450K Illumina platforms) to measure cord blood‐derived DNA methylation but obtained inconsistent findings (Adkins et al., 2011; Markunas et al., 2016). Both studies had less than 2000 individuals, and analytic methods used to control for blood cell type composition were not available at that time to account for the DNA from nucleated red blood cells using contemporary cord blood reference panels (Bakulski et al., 2016). Given the paucity of studies, we undertook a comprehensive investigation of the associations of parental age at birth with newborn and childhood DNA methylation in meta‐analyses of multiple datasets through the Pregnancy and Childhood Epigenetics (PACE) consortium (Felix et al., 2018).

## 2 METHODS

### 2.1 Study participants and data

Seventeen unique cohorts from PACE participated in the study: ALSPAC, CHAMACOS, CHS, DCHS, EAGeR, EARLI, Gen R, Gen3G, HELIX, INMA, MARBLES, MMNP, MoBa, NORTHPOP, PMMST, POSEIDON, and Upstate KIDS. Newborn or child blood samples were collected in each cohort and DNA methylation was obtained using the 450K or EPIC microarray (see Data S1 for cohort‐specific details).

### 2.2 Epigenome‐wide association study (EWAS) in newborns

All cohort analysts received an analytic plan specifying the models and covariates. References to “maternal/mothers” or “paternal/fathers” are taken to mean biological sex, with the female and male contributors of genetic material for conception, but may not be the gender the individuals identify with or a parental role that they serve. While cohort analysts used their own pipeline for initial quality control procedures such as beta value normalization as stipulated in the Data S1, all cohorts winsorized methylation data at the lower and upper 1% to control for extreme methylation values prior to running adjusted analyses using robust linear regression.

Each cohort analyst ran robust linear regression adjusting for covariates in three different models each for continuous maternal and paternal age, and then provided the results. The first model was minimally adjusted for cell type estimation (either using a cord blood reference for newborn data or using Houseman method if not), batch, and any selection criteria as applicable for the study. The second model (the main model of the study) added to that model the covariates of parental smoking, education, race/ethnicity, parity, and genetic principal components (as available, and in place of race/ethnicity where appropriate). Selection factors for three cohorts (HELIX, MMNP, and Upstate KIDS) were also adjusted for in their cohort‐specific analyses. If sample size for any single race/ethnicity category was less than 100, cohort analysts excluded them from analysis. BMI was added to the third model, owing to a drop in participants without that information for maternal (*n* = 1275, 13%) and paternal models (*n* = 1528, 19%). For paternal age models, if cohorts only had covariate information from mothers and not fathers (e.g., maternal smoking rather than paternal smoking), cohorts ran models using maternal information instead. Results from each model were restricted to probes shared in both the 450K and EPIC microarrays as over half of the data came from the 450K panel. For sensitivity analysis, infant sex was added to the main model as a technical covariate. However, given that the association of both maternal and paternal age on sex ratio has been observed (James & Grech, 2017; Zhao et al., 2023), potentially making infant sex a mediator, we selected our primary model without offspring sex as covariate.

### 2.3 Meta‐analyses

The *METAL* package in R (version 2020‐05‐05) was used to run fixed effects inverse variance weighted meta‐analysis (by R.J.B.). A shadow meta‐analysis of the main findings was run independently (by Y.H.) using *fastmeta* in R to exclude analytical errors. Multiple testing was accounted for by Benjamini‐Hochberg adjustment for false discovery rate (FDR) under each model/analysis (Benjamini & Hochberg, 1995). CpG sites on the sex chromosomes and cross‐reactive probes (Chen et al., 2013) were removed after FDR correction. A supplementary meta‐analysis was conducted on the EPIC probes, but this was considered exploratory as 53% of cohorts used the 450K microarray. Genomic inflation was evaluated using lambda values.

Similar analyses and meta‐analyses were conducted for child blood. However, due to the relatively smaller sample size of the childhood follow‐up methylation dataset, a look‐up for the CpGs identified in the EWAS using newborn DNA methylation was also conducted to evaluate the persistence of associations. We did not adjust for multiple testing in this assessment of the overlap between newborn and childhood blood DNAm, implementing a nominal significance level of 0.05.

### 2.4 Differentially methylated regions

Dmrff (version 1.0.0) was used to identify differentially methylated regions specifying a 500‐bp window as CpGs of a consecutive region (Suderman et al., 2018). Dmrff identifies two or more CpGs within this window as differentially methylated using inverse variance‐weighted meta‐analysis of the inputted effect sizes with multiple testing controlled by Bonferroni correction and accounting for correlation between CpGs. We inputted the meta‐analyzed results (estimates, standard deviations, *p*‐values) of the newborn blood DNAm differences with maternal age, adjusting for the main model covariates.

### 2.5 Functional characterization of the CpGs

CpGs were annotated to genes confirmed to occupy distances within 10,000 bp of any known gene using the UCSC Genome Browser. Pathways were interrogated using the missMethyl (Phipson et al., 2016) R package, taking the 217 CpGs at P_FDR_ < 0.20 from the main model for maternal age and newborn DNA methylation EWAS. Similarly, these 217 CpGs and the 33 CpGs (P_FDR_ < 0.05) were queried for tissues and transcription factors using eFORGE 2.0 and eFORGE‐TF (Breeze et al., 2019). There were too few CpGs associated with paternal age in the main model (14 at P_FDR_ <0.20) for meaningful bioinformatic analyses. Whether the EWAS‐identified CpGs impacted gene expression was cross‐checked using the HELIX publicly available database of autosomal cis‐ expression quantitative trait methylation (eQTM) sites in childhood blood samples, with adjustment for cell type estimates (Maitre et al., 2022; Ruiz‐Arenas et al., 2022). The catalog included methylation data from the 450K array compared with gene expression using the Human Transcriptome Array 2.0 on RNA from blood samples of over 800 children. Using data from the Genetics of DNA Methylation Consortium (GoDMC) catalog of >30,000 adults with blood, we also performed a look‐up of methylation quantitative trait loci (mQTLs) that may impact methylation at the identified CpGs (Min et al., 2021).

## 3 RESULTS

Fourteen cohorts contributed data from 9551 newborn samples (90% from cord blood and 10% from dried blood spots) for the maternal age meta‐analysis. Eleven of these cohorts also contributed data from 8162 newborn samples (89% from cord blood and 11% from dried blood spots) for the paternal age meta‐analysis. In addition, four cohorts contributed results derived from peripheral blood DNA methylation data from 2281 children (aged 5–10 years) for maternal age and 1559 children for paternal age meta‐analysis. Parental age distributions and their correlations are summarized in Table 1. Mean ages ranged from 26 to 34 years for mothers and 28 to 48 years for fathers. Women averaged 2–3 years younger than their male partners, with a mean correlation of 0.64 between maternal and paternal age (range 0.48–0.78). Few newborns were from teenage pregnancies (*n* = 6), with most cohorts excluding parents under the age of 18 years old. Most cohorts consisted of predominantly white Europeans except one consisting of mostly Mexican‐American Latino participants and two cohorts from India and Gambia. Additional cohort‐specific characteristics are summarized in Table S1.

**TABLE 1:** Parental age distributions by cohort.

Newborn cohorts	Maternal age					Paternal age					Correlation
	*N*	Min	Mean	SD	Max	*N*	Min	Mean	SD	Max	
ALSPAC	851	16	30	4	42	626	21	32	6	64	0.62
CHAMACOS	357	18	26	5	43	354	16	28	6	54	0.66
CHS	225	15	30	6	45	225	16	32	6	50	0.78
DCHS	248	18	27	6	42	—	—	—	—	—	—
EAGeR	358	19	28	4	39	—	—	—	—	—	—
EARLI	171	22	34	5	44	170	22	35	6	53	0.63
Gen R	1225	17	32	4	46	1203	16	34	5	58	0.68
Gen3G	440	19	29	4	48	—	—	—	—	—	—
INMA	385	18	30	4	42	385	19	32	5	51	0.67
MARBLES	238	21	34	5	47	234	21	37	6	56	0.68
MoBa1	897	18	30	4	43	897	18	32	5	57	0.62
MoBa2	570	18	30	4	44	570	19	33	5	49	0.69
MoBa4	841	18	30	5	43	841	21	33	5	58	0.67
MoBa8	1013	19	30	4	44	1013	20	33	5	64	0.68
NORTHPOP	722	19	30	4	43	706	19	32	5	69	0.62
POSEIDON	277	18	32	5	43	275	20	35	6	54	0.68
Upstate KIDS	733	17	32	6	48	663	19	34	7	61	0.75
Total/Average	9551	18	30	5	44	8162	19	33	6	57	0.67
Childhood Cohorts	*N*	Min	Mean	SD	Max	*N*	Min	Mean	SD	Max	
HELIX	1160	16	31	5	44	551	22	34	5	57	0.62
MMNP	682	17	27	5	40	610	19	33	5	49	0.69
PMMST	293	17	31	7	50	261	21	48	12	86	0.48
Upstate KIDS follow‐up	146	19	31	6	44	137	19	33	7	57	0.77
Total/Average	2281	17	30	6	45	1559	20	37	7	62	0.64

### 3.1 Maternal age EWAS identifies DNA methylation differences in offspring

**FIGURE 1:**
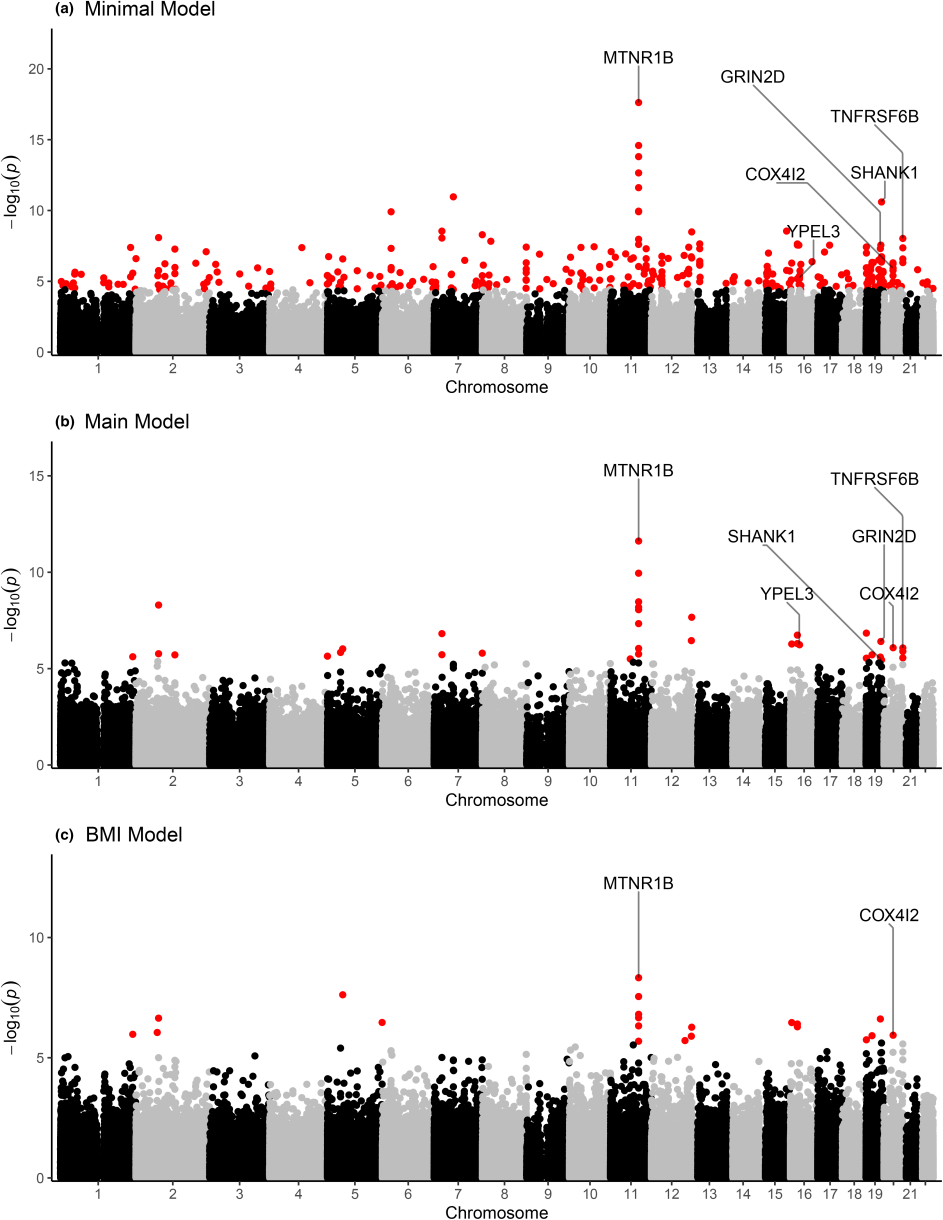
Manhattan plots for maternal age newborn blood EWAS. The Manhattan plots correspond to covariate adjustments for (a) cell type and batch, (b) additionally for maternal smoking, maternal education, maternal race/ethnicity/ancestry, parity, and selection (as applicable), and (c) additionally for BMI. Color indicates P_FDR_ < 0.05.

Figure 1 shows the Manhattan plots for maternal age in association with newborn blood DNA methylation for each of the covariate models. In the minimally adjusted model adjusted for batch and cell types, continuous maternal age was associated with differences (P_FDR_ < 0.05) in DNA methylation at 322 CpGs (Table S2). This number was reduced to 33 CpGs representing 13 loci (P_FDR_ < 0.05) in the main model as listed in Table 2 (with full annotation in Table S3). There was a tendency for associations to be in the positive direction, with only five of the 33 CpGs showing negative associations with maternal age. Among these 33 CpG sites, we found eight CpGs located on chromosome 11 in or near *MTNR1B*, all with positive associations, including the top hit at cg00528572. The difference presented (0.0013; SE 0.0002) translates to an increase of 0.67% (SE 0.1) DNA methylation per 5‐year increase in maternal age. Three of the 33 CpGs were located on chromosome 20 in *RTEL1‐TNFRSF6B* and were also positively associated with maternal age (effect sizes ranging 0.65%–0.78% per 5‐year increase in maternal age). To evaluate the robustness of the findings, we conducted a leave‐one‐out analysis. Figure S1 shows that no single cohort had a systematic influence on the meta‐analysis results.

**TABLE 2:** Maternal Age and Offspring Blood DNA methylation.

CpG^a^	Newborn blood DNA methylation							Childhood blood DNA methylation			
	Estimate	SE	*p*‐Value	Chr	Position	Gene	Direction^b^	Estimate	SE	*p*‐Value	Direction^c^
**cg00528572**	0.001341	0.000191	2.41E‐12	11	92,703,433	*MTNR1B*	++++++−+++−++++++	0.00097	0.00033	0.003	−+++
**cg07105285**	0.001011	0.000157	1.13E‐10	11	92,702,663	*MTNR1B*	+++++++−++−++++++	0.00034	0.0002	0.097	−−++
**cg03970229**	0.000626	0.000106	3.42E‐09	11	92,702,507	*MTNR1B*	+++++++++++++++++	0.00033	0.00015	0.027	−+++
**cg03292743**	−0.00095	0.000162	5.03E‐09	2	74,642,838	*C2orf81*	−−−−−−−−−−−−+−+−−	−0.00042	0.00027	0.129	−
**cg15559898**	0.000622	0.000107	6.53E‐09	11	92,703,185	*MTNR1B*	+++++++−++−++++++	0.00048	0.00018	0.008	−+++
**cg07609862**	0.001325	0.00023	8.77E‐09	11	92,702,912	*MTNR1B*	+++++++−++−++++++	0.00054	0.00024	0.023	−+++
**cg26352652**	0.000857	0.000153	2.17E‐08	12	133,013,600		++?++++−−−+++++++	0.00068	0.00025	0.007	++++
**cg01722932**	0.000608	0.000111	4.62E‐08	11	92,702,653	*MTNR1B*	+++++++−++−++++++	0.00026	0.00014	0.064	−+++
**cg12082025**	0.000674	0.000128	1.44E‐07	19	1,064,218	*ABCA7*	+++++−−−++−++++−+	−0.00001	0.00007	0.843	+−++
cg14898611	0.000684	0.00013	1.53E‐07	7	25,608,673		−++−+++++++++++++	0.00064	0.00022	0.003	−+++
**cg00538458**	0.000928	0.000178	1.83E‐07	16	22,959,868		−+++−++++++++++++	0.00072	0.0003	0.015	−+−+
**cg14503935**	0.000712	0.00014	3.53E‐07	12	131,864,894		−++−++++−?+++++++	0.00022	0.00024	0.372	+++−
cg12965344	0.000375	0.000074	3.91E‐07	19	48,898,160	*GRIN2D*	−++++++++−+++++++	0.00043	0.00014	0.002	+++−
**cg22968966**	0.00086	0.000171	5.04E‐07	16	22,959,875		++−+−++++++++++++	0.0008	0.00028	0.004	−+++
**cg07887168**	0.000561	0.000112	5.28E‐07	16	4,263,893	*SRL*	++++−+++++++−−++?	−0.00009	0.00015	0.563	+−−+
cg26709300	−0.00041	0.000082	5.80E‐07	16	30,106,682	*YPEL3*, *BOLA2B*	−−+−−−−+−−−+−+−−−	−0.00037	0.00014	0.008	−
**cg08918020**	0.000386	0.000078	8.25E‐07	20	30,225,706	*COX4I2*	−++++++−+++++++++	0.00041	0.00016	0.011	++++
cg16702083	0.001556	0.000316	8.46E‐07	20	62,328,427	*RTEL1‐TNFRSF6B*	++++−++−++++−++++	0.00229	0.00053	0.00001	++++
cg05803237	0.000508	0.000103	8.97E‐07	11	92,702,628	*MTNR1B*	++++++++++−++++++	0.00006	0.00014	0.642	−−++
**cg24536250**	0.000611	0.000125	9.43E‐07	5	50,218,625		+−−+−+++++++++++−	0.00021	0.00021	0.322	−+−+
cg24354818	0.001391	0.000287	1.27E‐06	20	62,328,094	*RTEL1‐TNFRSF6B*	++++−++−++++−++++	0.00177	0.00037	0.000002	++++
cg01817364	−0.00041	0.000084	1.45E‐06	5	43,037,411		−−−−−−++−+−−−−−−−	0.00005	0.0002	0.8	−−+−
cg24129222	−0.00097	0.000202	1.68E‐06	2	74,643,251	*C2orf81*	−−−−−−−−−−−−+−−−−	−0.00039	0.00036	0.279	−
cg12600858	0.000643	0.000134	1.76E‐06	11	92,702,530	*MTNR1B*	+++++++++++++++−+	0.00049	0.00023	0.03	−+++
cg13728287	0.000626	0.000131	1.90E‐06	7	25,608,634		++−−++++++−++++++	0.00059	0.00021	0.005	−+++
**cg09405380**	0.000453	0.000095	1.93E‐06	19	19,730,109	*PBX4*	−+++++−−+++++++++	−0.00003	0.00013	0.817	−+−−
cg06741367	0.000857	0.00018	1.94E‐06	2	128,453,108		+++++++++++++++++	0.00053	0.00023	0.022	++++
**cg24488001**	0.00053	0.000112	2.42E‐06	1	238,644,629	*LINCO1139*	++−+−+−−+++++?+??	−0.00004	0.00016	0.79	+−−+
**cg18473455**	0.000755	0.00016	2.52E‐06	19	47,333,922	*SNAR‐E*	++−+++++++++−++++	−0.00013	0.00032	0.681	−+−−
cg23773946	0.001299	0.000277	2.76E‐06	20	62,327,968	*RTEL1‐TNFRSF6B*	++++−++−+−++++++?	0.00242	0.00062	0.00009	++++
cg05372495	0.000413	0.000088	2.88E‐06	19	1,063,624	*ABCA7*	++−+++−++++++++++	0.00002	0.00007	0.719	+−++
cg15829826	−0.00034	0.000072	3.13E‐06	11	65,153,860	*FRMD8*	−−−−−−+−−−−−+−−−−	−0.00011	0.00007	0.125	−−−+
cg25594486	0.000589	0.000127	3.51E‐06	19	51,165,441	*SHANK1*	++++−++++++++++++	0.00073	0.00029	0.013	−++−

*Note*: The 33 CpGs associated at birth (P_FDR_ < 0.05) with original *p*‐values shown above. Maternal age was modeled in years and adjusted for cell types, maternal smoking, maternal education, maternal race/ethnicity/ancestry, parity, batch, and selection (as applicable, see Data S1). These 33 CpGs were also reviewed in childhood analyses adjusting for the same covariates. Estimates represent the difference in DNA methylation levels (i.e., beta values) per year of maternal age. See Table S3 for full annotation including location information and heterogeneity statistics.

Abbreviations: Chr, chromosome; SE, standard error.

^a^ Bolding indicates remained significant (P_FDR_ < 0.05) in model 3 (after additional adjustment for maternal BMI).

^b^ Cohorts listed in the following order for newborn associations: EAGeR, Gen3G, DCHS, ALSPAC, NORTHPOP, GenR, POSEIDON, EARLI, MARBLES, CHAMACOS, INMA, MoBa1, MoBa2, MoBa4, MoBa8, Upstate KIDS, and CHS.

^c^ Cohorts listed in the following order for childhood associations: Upstate KIDS, HELIX, MMNP, and PMMST.

Maternal BMI was added as a covariate due to its impact on fecundability (Gesink Law et al., 2007), which can increase maternal age at delivery. We sequentially added BMI because further adjustment diminished the sample size by ~11% fewer participants due to missing BMI information. Twenty‐one CpGs were identified in these models (Table S4). Of these, the 18 overlapping CpGs between the main model and the BMI model are bolded in Table 2 and includes six of the eight *MTNR1B* CpGs but not the three *RTEL1‐TNFRSF6B* CpGs. Figure S2 shows the volcano plots for all three newborn blood DNA methylation models (with lambdas 1.10, 1.09, and 1.06 for models 1, 2, and 3, respectively).

We also conducted differentially methylated region (DMR) analysis of the main model results (not adjusted for BMI) using dmrff, an analysis procedure that accounts for the correlation between nearby CpGs (Suderman et al., 2018). Seven regions were identified by dmrff to be associated with maternal age (Table 3). Corroborating the EWAS results, a region of nine CpGs spanning 926 bp annotated to *MTNR1B* on chromosome 11 (P_FDR_ = 6.92 × 10^−8^) showed significantly higher methylation with advancing maternal age. Three of these nine CpGs are located in the *MTNR1B* gene body, one in the first exon, and the remaining six within 1500 bp prior to the transcription start site (Figure S3). The regional analysis also identified a region of four CpGs in/near *SHANK1*, which also had higher methylation with greater maternal age. The remaining five regions consisted of 2–3 CpGs, and two were located in/near a known gene region (i.e., *C2of81* and *BOLA2B/YPEL3*).

**TABLE 3:** Maternal age and differentially methylated regions (DMRs) in newborns.

Region	Chr^a^	Start^a^	End^a^	*n*	B	S	Raw *p*‐value	Bonferroni *p*‐value^b^	Gene	Corresponding CpGs
1	11	92,702,507	92,703,433	9	0.016275	0.002202	1.47E‐13	6.92E‐08	*MTNR1B*	*cg03970229* *cg12600858* *cg05803237* *cg15842276* *cg01722932* *cg07105285* *cg07609862* *cg15559898* *cg00528572*
2	2	74,642,838	74,643,251	2	−0.01542	0.002702	1.15E‐08	0.0054	*C2of81*	*cg03292743* *cg24129222*
3	16	30,106,682	30,106,897	3	−0.00766	0.001365	1.99E‐08	0.0094	*BOLA2P/YPEL3*	*cg26709300* *cg16348385* *cg27106909*
4	16	22,959,819	22,959,875	3	0.017781	0.003223	3.46E‐08	0.0162		cg08259413 cg00538458 cg22968966
5	12	133,013,600	133,013,765	2	0.011577	0.002099	3.47E‐08	0.0163		*cg26352652* *cg08634598*
6	19	51,165,441	51,165,845	4	0.007246	0.001341	6.62E‐08	0.0311	*SHANK1*	*cg25594486* *cg27277859* *cg00380835* *cg23374892*
7	7	25,608,634	25,608,673	2	0.010928	0.002036	8.05E‐08	0.0378		*cg13728287* *cg14898611*

*Note*: Using results from the main model adjusted for cell types, maternal smoking, maternal education, maternal race/ethnicity/ancestry, parity, batch, and selection (where applicable).

^a^ Chromosome location with position start and end (hg19).

^b^ Adjusted for multiple testing by Bonferroni correction (DMRff analysis using 500 bp window).

We then compared the findings at CpGs identified in the EWAS of maternal age and newborn blood DNA methylation with the EWAS of maternal age in relation to childhood blood DNA methylation, both adjusted for the main model covariates (Table 2). For the identified CpGs in the *MTNR1B* gene, we found that greater maternal age was associated with higher DNA methylation in childhood blood cells (P_nominal_ < 0.05, not accounting for multiple testing), in line with the direction of associations noted in the newborn blood DNA methylation. The three CpGs in the *RTEL1‐TNFRSF6B* gene identified at birth also remained consistent in direction (P_nominal_ < 0.0001) and had larger effect sizes in the childhood blood EWAS. For instance, per 5‐years of maternal age, DNA methylation of cg16702083 in *RTEL1‐TNFRSF6B* was associated with increases of 0.78% in DNAm at birth and of 1.15% in childhood. Of the other 33 CpGs identified from the newborn analysis, 10 others were also nominally significant in childhood blood, mapping to four known loci (i.e., *GRIN2D*, *YPEL3/BOLA2B*, *COX4l2*, *and SHANK1*). Lastly, the childhood meta‐analysis also identified three CpGs in/near *PRR25* on chromosome 16, which showed lower blood DNA methylation with greater maternal age (P_FDR_ < 0.05; Table S5).

### 3.2 Paternal age EWAS did not identify DNA methylation differences in offspring

The paternal age EWAS meta‐analysis included 8162 newborns (86% of those contributing to the above maternal age results) with blood DNA methylation information. Paternal age was associated with 11 CpGs in the minimally adjusted model, two CpGs in the main model, and seven CpGs in the BMI adjusted model (P_FDR_ < 0.05). (Table 4). Associations tended to indicate lower methylation with increasing year of paternal age, but none of the CpGs overlapped between all three models. The four CpGs that did overlap between any two models are indicated by their paired superscripts; two of these were in *RTEL1‐TNFRSF6B* (cg24354818 and cg07620230). These two CpGs were borderline significant with similar effect sizes in the main model (P_FDR_ = 0.07). It is difficult to surmise, but potentially the correlation between maternal and paternal age (mean of 0.64 across all cohorts) may explain some of these *RTEL1‐TNFRSF6B* associations, as a comparison of their effect sizes suggests that maternal associations were somewhat stronger than paternal associations at these *RTEL1‐TNFRSF6B* CpGs (e.g., looking again at cg16702083, 5‐years of paternal age was associated with 0.65% DNAm increase at birth and 1.02% in childhood). Tables S6–S8 provide further information on results from each of these models.

**TABLE 4:** Paternal age and offspring blood DNA methylation.

CpG	Newborn blood DNA methylation							Childhood blood DNA methylation			
	Effect	SE	*p*‐Value	Chr	Position	Gene	Direction^a^	Effect	SE	*p*‐Value	Direction^b^
Min. model								Main model			
cg21005510^a^	−0.00077	0.000126	1.08E‐09	7	63,353,570		−				
cg24354818^b^	0.001192	0.000223	9.06E‐08	20	62,328,094	*RTEL1‐TNFRSF6B*	++++−+++++++++	0.001207	0.000314	0.00012	++++
cg07620230^c^	0.001099	0.000216	3.41E‐07	20	62,328,084	*RTEL1‐TNFRSF6B*	+−++−+++++++++	0.001151	0.000307	0.00018	++++
cg20110801	−0.00052	0.000102	4.07E‐07	19	56,014,812	*SSC5D*	−−−+−−−−−−−−−−				
cg23548885	0.000137	2.73E‐05	5.06E‐07	2	47,150	*FAM110C*	++++++++++++−+				
cg05282518	−0.00066	0.000132	5.09E‐07	14	20,344,920	*OR4K2*	−−−−−−−−−−−−+−				
cg17900015	−0.00051	0.000102	5.76E‐07	17	38,957,507	*KRT28*	−−−+−+++−−−−+−				
cg14583127	−0.00072	0.000146	7.86E‐07	10	37,940,493		−−−−+−−−−−−−−−				
cg07105285	0.000611	0.000124	8.77E‐07	11	92,702,663	*MTNR1B*	+++−++++++++++				
cg16702083	0.001308	0.000266	8.89E‐07	20	62,328,427	*RTEL1‐TNFRSF6B*	++++−+++++++++	0.002056	0.000446	3.96E‐06	++++
cg07103517	−0.0004	8.32E‐05	1.29E‐06	7	50,348,485	*IKZF1*	−				
Main model								Main Model			
cg21005510^a^	−0.00074	0.00014	1.37E‐07	7	63,353,570		−−−+−+−−−−−−−−				
cg15893204^d^	−0.00017	3.24E‐05	1.62E‐07	1	56,247,952		−−−−−−+−−−−−−?				
BMI model								Main Model			
cg05244581	−0.00044	8.30E‐05	8.87E‐08	8	25,315,002	*PPP2R2A; KCTD9; CDCA2*	−				
cg03772384	−0.0003	5.63E‐05	1.17E‐07	3	12,851,369	*CAND2*	−−−−+−−−−−−−				
cg15893204^d^	−0.00018	3.48E‐05	1.58E‐07	1	56,247,952		−−−−−+−−−−−−				
cg11595575	0.001026	0.0002	2.71E‐07	5	140,235,510	*PCDHA1‐PCDHA10*	+++++?+????+	0.000734	0.000248	0.0031	++++
cg00227110	−0.00025	4.84E‐05	3.01E‐07	11	124,056,297		−−−++−−−−−−−	0.000145	7.25E‐05	0.045	−+++
cg24354818^b^	0.001362	0.00027	4.46E‐07	20	62,328,094	*RTEL1‐TNFRSF6B*	+++−++++++++	0.001207	0.000314	0.00012	++++
cg07620230^c^	0.001295	0.000259	6.04E‐07	20	62,328,084	*RTEL1‐TNFRSF6B*	+++−++++++++	0.001151	0.000307	0.00018	++++

*Note*: For newborn results, paternal age modeled in years and minimal model adjusted for cell types and batch; main model additionally adjusted for maternal smoking, maternal education, maternal race/ethnicity/ancestry, parity, and selection (as applicable, see Data S1); BMI model additionally adjusted for BMI. Results noted if P_FDR_ < 0.05. For childhood results, only the main model was used and included only nominally significant (*p* < 0.05) results. * Superscripted letters a,b,c,d designates the same CpG identifed across the adjusted models.

^a^ Cohorts listed in the following order for newborn models: ALSPAC, NORTHPOP, GenR, POSEIDON, EARLI, MARBLES, CHAMACOS, INMA, MoBa1, MoBa2, MoBa4, MoBa8, UKIDS, CHS; BMI model excluded one cohort for missing BMI information (NORTHPOP) but was otherwise in the same order.

^b^ Cohorts listed in the following order for childhood models: Upstate KIDS, HELIX, MMNP, PMMST.

The 16 unique CpGs identified by newborn analyses were also reviewed for childhood blood DNA methylation associations with paternal age (from the main model). As it was a much smaller sample size (*n* = 1559), nominal significance (*p* < 0.05) was used to generate the table. Only five unique CpGs overlapped with childhood blood DNA methylation at *p* < 0.05, and all but one CpG (cg00227110) would be significant even with Bonferroni correction for 16 lookups (0.05/16 = 0.003). Three CpGs were in *RTEL1‐TNFRSF6B*, and one CpG was in *PCDHA1‐10*. Table S9 shows the meta‐analyzed results of the full EWAS for paternal age on childhood blood methylation. Only one CpG was identified as FDR‐significant. Lambda values ranged 1.07–1.15 in paternal age meta‐analyses (Table S10).

### 3.3 Supplemental meta‐analysis of EPIC microarray data

The current meta‐analyses included DNA methylation data at the 450K probes measured by cohorts common to both arrays (the 450K or EPIC microarrays). Results associated with maternal age in newborns were similar in direction using the EPIC array as the 450K. A supplemental meta‐analysis of maternal age with newborn blood DNA methylation using only cohorts with EPIC microarray data (*n* = 4323, 47%) did not identify any CpG sites that reached the FDR significance threshold; neither did a meta‐analysis of paternal age. For data sharing, full lists of associations that were nominally significant (*p* < 0.05) in EWAS meta‐analyses is provided on Figshare including analyses with the EPIC array (as listed in Table S11).

### 3.4 Sensitivity meta‐analysis adjusting for infant sex

For maternal age with newborn DNA methylation, adjusting for infant sex identified 48 CpGs, including 32 of the original 33 CpGs. cg15829826 (in/near *FRMD8*) became borderline FDR‐significant (*p* = 0.06). All had similar effect sizes as shown previously without sex adjustment in Table 2. Of the 16 new CpGs identified, seven were not located in/near gene, and five had been mentioned previously as part of the DMRs they belong to (e.g., MTNR1B, SHANK1, etc) or as part of paternal age analysis (i.e., *RTEL1‐TNFRSF6B*). DMR analysis with the output also identified the same set of DMRs. For paternal age with newborn DNA methylation, the two CpGs identified (cg21005510, cg15893204) became borderline significant (P_FDR_ = 0.06). Results for both maternal and paternal age and childhood DNA methylation look‐ups were also similar and did not change particularly for the CpGs in/near MTNR1B or RTEL1‐TNFRSF6B. The full output is available on FigShare (Table S11).

### 3.5 In silico functional analyses

#### 3.5.1 Enrichment analyses for gene‐sets, tissues, and transcription factors

We performed Gene Ontology (GO) and KEGG pathway analyses on 217 CpGs (154 of which mapped to 132 annotated genes) at P_FDR_ <0.20 from the maternal age and newborn DNA methylation results (adjusting for main model covariates) using missMethyl (Phipson et al., 2016). We did not find any significant enrichment (Table S12). We then used the eFORGE database (Breeze et al., 2019), which curates epigenetic information, to understand the potential cell‐specific landscape of these CpGs. H3K4me1 cell‐specific enrichment was detected in fetal adrenal gland (*q* < 0.01), neural progenitor cells (*q* < 0.05), and fibroblasts (*q* < 0.05). No enrichment was detected for DNase1 hotspots or for chromatin states (Figure S4). There was no enrichment detected on any track when using a list of only the 33 FDR‐significant CpGs in eFORGE analysis (data not shown).

Using the eFORGE‐TF database, co‐location of CpGs with transcription factor (TF) binding sites was examined. Table S13 shows a table of the 33 CpGs associated with maternal age (in newborn main models) with the presence of transcription binding sites in fetal tissues (including placenta). The gene‐specific transcription factor motifs were also summarized by searching the eForge‐TF database using the nine CpG probes of *MTNR1B* DMR. The top motif for identified CpGs at *MTNR1B* was for the PAX (paired box; PAX5) transcriptional regulator, which is highly conserved, present across tissue types, and plays critical roles in development (Mayran et al., 2015).

#### 3.5.2 Expression quantitative trait methylation

We conducted a review of the significant CpGs in previously published blood DNA methylation and gene expression associations in children from the HELIX project (Maitre et al., 2022; Ruiz‐Arenas et al., 2022). Three out of the 33 CpGs from the main model (cg26709300, cg01817364, and cg05372495) were associated with expression of several nearby genes, including *ITGAL*, *ANXA2R*, *CCL28*, *WDR18*, *and ABCA7* (Table S14). One of these CpGs (cg26709300) was part of a differentially methylated region identified on chromosome 16 at *BOLA2B/YPEL3*. The other 30 CpGs were not associated with expression levels in the HELIX cohort's childhood blood samples.

#### 3.5.3 SNP influences on blood DNA methylation associations at identified CpGs

While we excluded infants with chromosomal abnormalities from analysis, the question remained if the associations observed were driven by known common SNP relationships with DNA methylation at these loci. We conducted a look‐up of methylation quantitative trait loci (mQTL) that may influence the blood DNA methylation levels at the 33 CpGs identified by the analyses between maternal age and newborn blood DNAm (from the main model). Using the GoDMC catalog (Min et al., 2021) of over 30,000 adults, we compiled the numbers of potential mQTLs associated with each of the 33 CpGs (Table S15). Few SNP associations were traced to each *MTNR1B* CpG (<10). However, there were numerous SNPs associated with methylation at nearly all other CpGs, including two CpGs at the *RTEL‐TNFRSF6B* gene (cg16702083, cg24354818).

## 4 DISCUSSION

In this meta‐analysis of blood DNAm levels from over 9500 newborns and 2000 children, maternal rather than paternal age was associated with differential DNAm at 33 CpGs. CpGs near/in *MTNR1B* and *RTEL1‐TNFRSF6B* were associated with increasing older maternal age at delivery. Moreover, the CpGs located in *RTEL1‐TNFRSF6B* were consistent at birth and in childhood. Multiple transcription factor binding sites were proximate to the *MTNR1B* and *RTEL1‐TNFRSF6B* CpGs. In addition, three of the 33 CpGs (cg26709300, cg01817364, and cg05372495) were associated with expression levels at various loci including *ITGAL*. In line with individual CpG findings, a differentially methylated region (DMR) consisting of nine CpGs near/in *MTNR1B* was also identified, where older maternal age at delivery was associated with higher DNAm in newborn blood. A DMR consisting of four CpGs in *SHANK1* was also identified. Our findings suggest that DNA methylation may explain the intergenerational connection observed linking older maternal age and long‐term offspring health.

The exact mechanisms to inter−/trans‐generational inheritance of epigenetic marks remain largely unknown but are suspected to vary by exposure. Observations on how maternal age affects DNA methylation in oocytes have been noted in the literature, which can shed light on maternal‐age‐related differences in offspring phenotypes (Castillo‐Fernandez et al., 2020). In a single‐cell analysis of natural cycle oocytes from young and old mice (12 vs. 45+ weeks old), distinct distributions of transcripts and patterns of DNA methylation emerged. First, transcript abundance measured by scRNA‐seq was lower in oocytes from older female compared to younger female mice. Lower CpG DNA methylation but higher non‐CpG (uniquely abundant in oocytes) DNA methylation was also observed in the aged oocytes compared to young ones, even after making subgroup comparisons in oocytes deemed more developmentally competent. Yet other studies have found reduced expression of genes found to be involved with DNA methylation establishment and maintenance (i.e., DNMTs) with concomitant decline in methylation with age. (Reviewed in Klutstein & Gonen, 2023) Differences in resulting murine embryos were also observed. Exact mechanisms of how these DNAm differences in oocytes may perpetuate to the human embryo are unclear but recent evidence substantiates downstream perpetuation despite the well‐recognized erasure and re‐establishment of DNA methylation at fertilization. Particularly, in a single‐cell analysis of human chorionic villi samples, lower DNA methylation of several DMRs including for the GNE gene which corresponded with lower transcription that could potentially explain the higher risk of spontaneous abortion with maternal age (Qin et al., 2023). Whereas the reviewed evidence suggests lower global methylation with maternal age, we observed higher DNA methylation at most of the single‐site CpGs identified. Hence, alternative transgenerational mechanisms apart from lower oocyte DNMTs and transcript abundance may play a role, including non‐coding RNA, changes in the 3D genome structure, and transcription factor binding (Reviewed in Fitz‐James & Cavalli, 2022).

To place these findings in context, we considered the biological relevance of *MTNR1B* and *RTEL1‐TNFRSF6B* in greater detail from the literature. *MTNR1B* codes for a transmembrane receptor of melatonin, and its mutations have been associated with wide‐ranging health effects including type 2 diabetes (Zhu et al., 2023) and sleep and neuropsychiatric disorders (Comai & Gobbi, 2014), conditions that are also more frequent among offspring of older mothers (Zhang et al., 2022). Some evidence suggests DNA methylation at *MTNR1B* in peripheral blood mononuclear cells correlates with lower melatonin levels (Lesicka et al., 2023), although we did not find direct correlation with gene expression in a look‐up of HELIX data for childhood blood. Nevertheless, several transcription factor motifs were proximate to the CpGs identified across tissue types, including the PAX (paired box; PAX2/5/9) transcriptional regulators, whose mutations are associated with multiple neurodevelopmental disorders (Mayran et al., 2015). Even if the small shifts in DNA methylation do not translate to differences in gene expression of melatonin or other proteins, they may serve as a biomarker pointing to the inheritance of circadian rhythm differences that may have long‐term consequences. The heritability of sleep, for instance, is difficult to quantify given the difficulties in measurement of sleep and sleep quality (Lewis & Gregory, 2021). Evidence also suggests that melatonin acts through epigenetic mechanisms including via DNA methyltransferases (Li et al., 2020; Linowiecka et al., 2023).

The *RTEL1‐TNFRSF6B* locus consists of two naturally occurring transcripts neighboring each other. *RTEL1* (regulator of telomere elongation helicase 1) is a DNA helicase maintaining genome integrity (Hourvitz et al., 2023) and whose mutations have been identified with shorter leukocyte telomere length, a measure of biological age (Dorajoo et al., 2019). *TNFRSF6B* codes for tumor necrosis factor receptor superfamily, member 6b, or also referred to as decoy receptor 3, DcR3, which plays a role in inflammatory pathways. Read‐through transcription from *RTEL1* into *TNFRSF6B* combined results in a non‐coding RNA. Methylation of *TNFRSF6B* is associated with allergies (Imran et al., 2022). One study evaluated naïve CD4 T cell‐specific methylation using the EPIC micro‐array in blood samples of adolescents with and without an IgE‐mediated food allergy, finding higher methylation in the allergy group for a DMR of seven probes (Imran et al., 2022). Childhood allergic rhinitis and food allergies have been associated with older maternal age (Lu et al., 2020), which may be mediated by these differences in methylation observed among newborns. In our analysis, *RTEL1‐TNFRSF6B* methylation levels were positively correlated with both maternal and paternal age. Thus, the specificity of the association remains unclear, as mutual adjustment was not conducted due to collinearity concerns.

Besides these two loci, offspring DNA methylation at CpGs in 11 other loci were associated with maternal aging at birth. CpG associations in 4 loci were persistent through childhood (*GRIN2D*, *YPEL3/BOLA2B*, *COX4l2*, *SHANK1*), and one CpG (cg26709300 at *YPEL3/BOLA2B*) was reported in a catalog of cis‐eQTMs as associated with the expression of *ITGAL*, which forms the alpha integrin involved in leukocyte intercellular adhesion (Lefort & Ley, 2012). Further biologic information on the three DMRs identified at *SHANK1*, *C2orf81 and BOLA2B/YPEL3* are in Data S1 after the section on cohort descriptions.

The two previous studies that evaluated newborn offspring DNA methylation with respect to parental age using microarray technology (Adkins et al., 2011; Markunas et al., 2016) did not identify the same set of CpGs as we observed. The first study examined DNA methylation using the Illumina 27 K Beadchip (which contains much fewer probes compared to the 450K and EPIC arrays) among 168 newborns and identified 144 CpGs by Spearman correlations (Adkins et al., 2011). They also found it difficult to tease apart the impacts of maternal and paternal age due to their correlation (*r* = 0.75). Another study using 450K data from the Norway Facial Clefts Study (NFCS; 418 cleft cases, 480 controls) found lower DNA methylation at four adjacent CpGs in/near *KLHL35* with advancing maternal age (Markunas et al., 2016). All four CpGs were replicated in the Norwegian Mother, Father, and Child Cohort Study (MoBa) (*n* = 1062), albeit with smaller effect sizes; they found no associations with paternal age. Offspring DNAm at these four CpGs in/near *KLHL35* were not even nominally associated (all *p* < 0.05) with maternal age in the current analysis. Both papers were published prior to an updated cell type reference (Gervin et al., 2019), which could explain the inconsistent findings. Another study evaluated 2740 adult daughters, finding maternal age was associated with differential methylation at 87 CpGs, nine of which occurred in the promoter region of *LHX8*, integral in female fertility (Moore et al., 2019). However, our investigation is not comparable as the daughters of that study were over 35 years old (average 57 years) at time of blood methylation assessment.

Studies of aging and DNA methylation were also highlighted by the EWAS catalog. In particular, a previous study on DNA methylation, with some overlapping newborn data supplied from two cohorts in the current analysis (ALSPAC and Gen R), investigated variation from birth to adolescence using trajectory analyses (Mulder et al., 2021). The study highlighted that 51.6% of CpGs changed as children age (particularly with decreasing methylation over time). As the majority of CpGs were discovered to change, almost all 33 found here to be associated with maternal age were among them. In a look‐up of their publicly shared database, the *MTNR1B* CpGs exhibited large inter‐individual variability with a few increasing in methylation over time but many staying relatively stable. This trajectory may align with why they were also identified in childhood despite being from vastly different cohorts and again speaks to the enduring impact of maternal age. Five other CpGs were previously found to be associated with gestational age (cg14898611, cg07887168, cg13728287, cg09405380, and cg15829826) (Hannon et al., 2019; Kashima et al., 2021; Spiers et al., 2015).

We recognize difficulties in teasing apart the downstream (mediating) impact of infertility and underlying chronic or pregnancy conditions that accumulate with age. However, we did not adjust for these mediators on the causal pathway to avoid overadjustment bias (Schisterman et al., 2009). Nevertheless, previous EWAS findings were queried in PubMed along with the EWAS Catalog (Battram et al., 2022) and EWAS Atlas (Li et al., 2019) to evaluate whether the *MTNR1B* and *RTEL1‐TNFRSF6B* CpGs were related to fertility and pregnancy conditions. The only study of relevance was from the MoBa cohort, which observed lower DNA methylation of eight CpGs at *MTNR1B* between newborns conceived by assisted reproductive technologies (ART) compared to those not (Haberg et al., 2022). Given the opposing direction of association from what is observed in the current analysis (i.e., maternal age was associated with higher, rather than lower, DNA methylation), it is unlikely that ART mediated the observed associations. Furthermore, many cohorts excluded newborns conceived by ART, and the current meta‐analysis did not include the large ART comparison group from MoBa's previous publication (see Data S1 for cohort details).

### 4.1 Study limitations

While we adjusted for education, the socioeconomic advantage of being older parents was likely incompletely captured. We suspect that the social impact of parental age is greater in childhood than at birth, given postnatal benefits such as sufficient resources (financial or otherwise) playing a role in later life. That the *MTNR1B* and *RTEL1‐TNFRSF6B* associations also persisted in childhood among cohorts suspected to have wholly different confounding structures (i.e., postnatal data from India and The Gambia) strengthens robustness of those findings. The microarrays used covered a fraction of the whole genome, and all analyses were conducted using blood sources of DNA, which may not represent tissue‐specific differences. Effect sizes were small, even after translating to 5‐year age differences in % DNA methylation (<1%). However, we cannot rule out that large effect sizes to one cell type consisting of a small fraction analyzed were present as we were unable to implement cell‐specific analyses. Moreover, in vivo studies specifically showing differences in protein levels could not be conducted to corroborate functionality. However, catalog searches pointed to associations with transcription factors and gene expression that may still play a role despite small effect sizes, and such small effect sizes in research for understanding the Developmental Origins of Health and Disease hypothesis is not uncommon (Breton et al., 2017).

## 5 CONCLUSION

We found differences in offspring DNA methylation at several CpG sites and genomic regions in relation to older maternal age, which may explain some of the observed associations with long‐term health outcomes in offspring. The lack of associations with paternal age remains to be deciphered in future work, requiring greater inclusion of fathers in research.

## AUTHOR CONTRIBUTIONS

EY conceived and designed the study, drafted the initial manuscript, designed and oversaw the acquisition of DNA methylation data in Upstate KIDS cohorts. RJB conducted the meta‐analysis and bioinformatic analysis. YH conducted the shadow meta‐analysis. JR contributed to the analysis plan and script. JF and SL designed and oversaw the PACE consortium. CMP, LCH, FMB, GM, HRE, SRM, RI, PI, CA, JH, DK, EP, ZN, JD, EC, IFM, CA, and AH analyzed results in their respective cohorts and drafted cohort‐specific summaries. WG, GRC, DS, SLM, SHW, AC, RR, LB, and SS provided substantial contributions to the interpretation of the results. VWVJ, JS, EFS, JW, RG, KBG, AP, DJS, HJZ, RS, DF, CB, TS, BE, SAS, MD, and CW established their respective cohorts for phenotypic data collection. NH, LZ, JF, CHDF, MJS, MV, MB, SL, GS, MFH, and SH designed and oversaw the acquisition and analyses of DNA methylation data in their respective cohorts. All authors critically revised the manuscript and approved the final manuscript.

## FUNDING INFORMATION

EY was supported by the Intramural Research Program of the *Eunice Kennedy Shriver* National Institute of Child Health and Human Development. The Upstate KIDS and EAGeR studies were funded under contract numbers: HHSN267200603423, HHSN267200603424, HHSN267200603426, HHSN275201300023I, HHSN2750008, HHSN275201200005C, HHSN267200700019C, HHSN275201400013C, HHSN275201300026I/27500004, HHSN275201300023I/27500017.

## CONFLICT OF INTEREST STATEMENT

None to declare.

## Supporting information


Figure S1.

Figure S2.

Figure S3.

Figure S4.

Tables S1–S15.

Data S1.

## Data Availability

The meta‐analyzed data that support the findings of this study are available on Figshare. The data availability of each participating cohort is described in the Supplemental Materials.
