# Supplementary figures and images for: Maternal age is related to offspring DNA methylation: A meta‐analysis of results from the PACE consortium
Source: Aging Cell. 2024 May 29;23(8):e14194. doi: 10.1111/acel.14194 (PMC11320347; doi:10.1111/acel.14194)

cg00528572

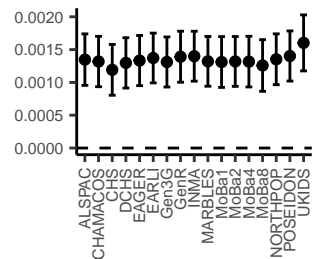

cg00538458

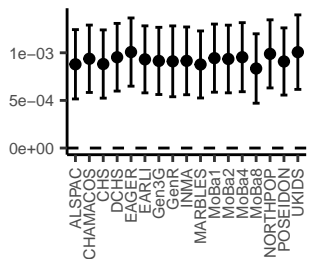

cg01722932

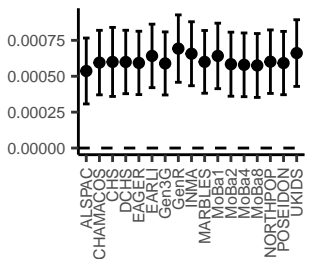

cg01817364

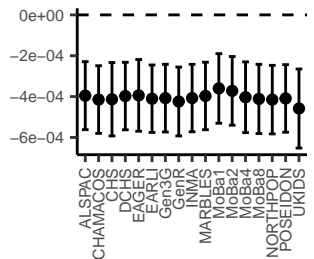

cg03292743

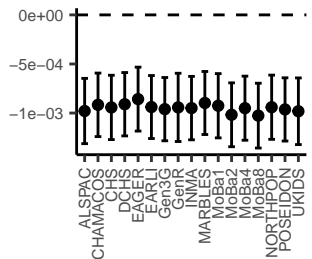

cg03970229

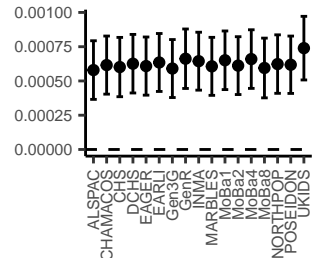

cg05372495

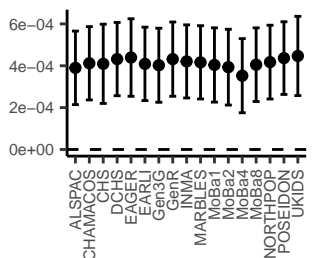

cg05803237

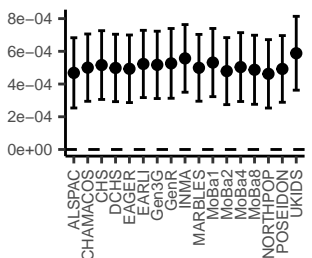

cg06741367

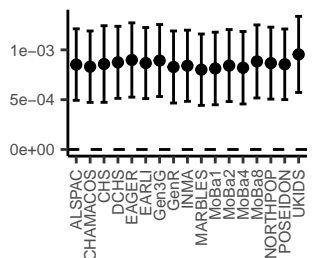

cg07105285

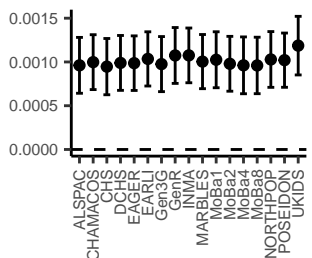

cg07609862

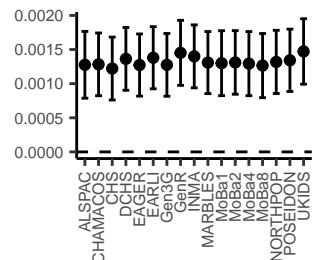

cg07887168

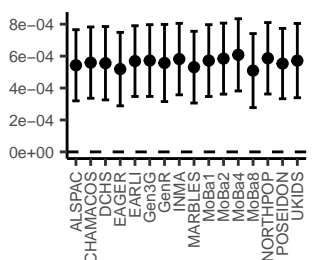

cg08918020

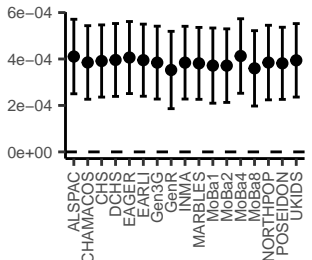

cg09405380

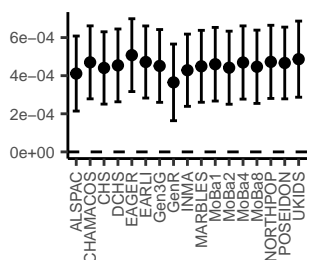

cg12082025

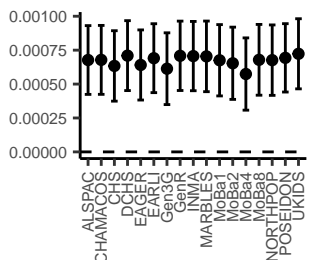

cg12600858

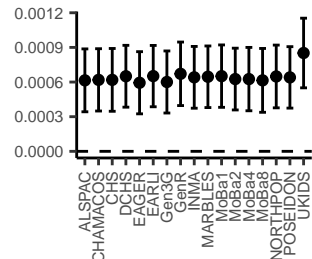

cg12965344

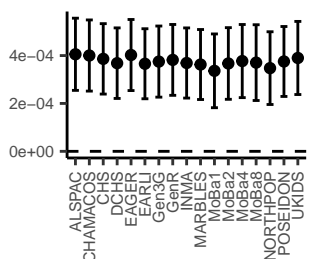

cg13728287

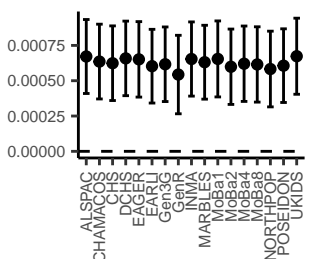

cg14503935

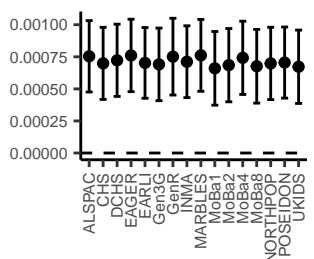

cg14898611

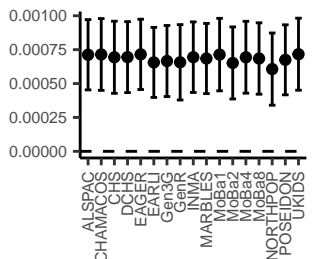

cg15559898

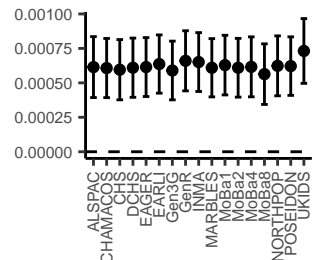

cg15829826

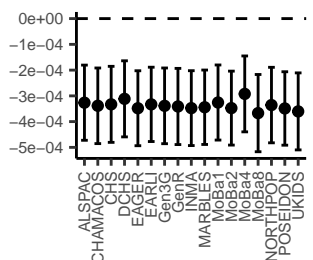

cg16702083

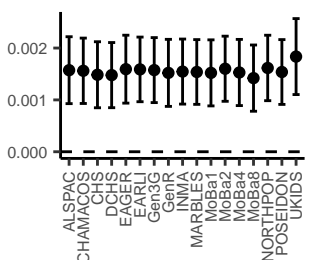

cg18473455

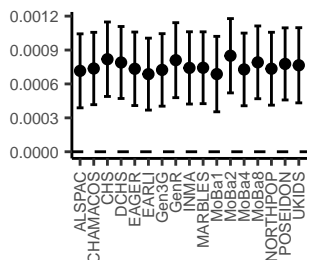

cg22968966

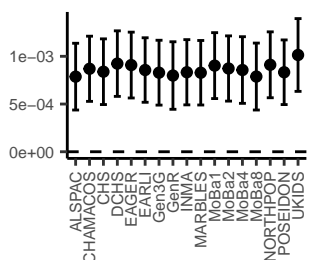

cg23773946

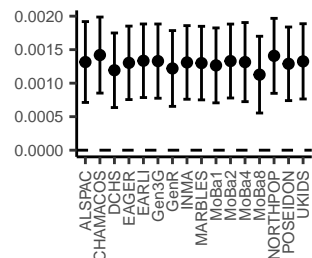

cg24129222

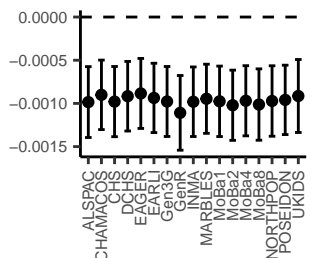

cg24354818

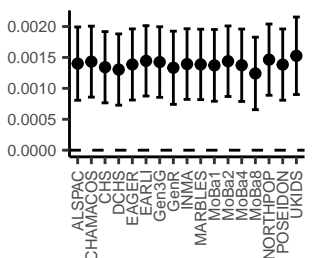

cg24488001

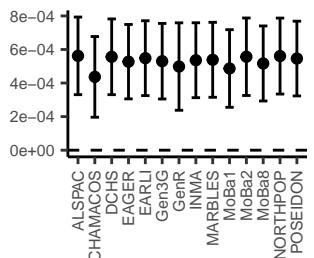

cg24536250

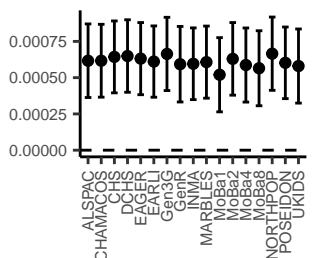

cg25594486

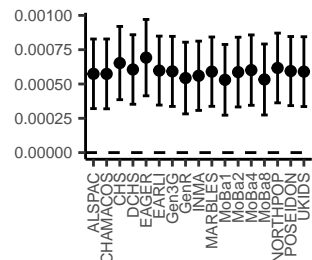

cg26352652

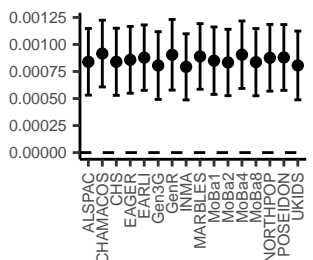

cg26709300

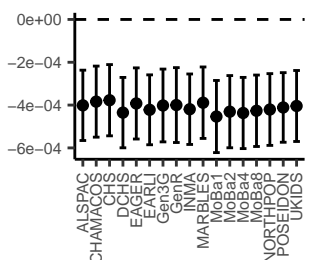

Supplement: Supplementary file 1 — Figure S1. [file ACEL-23-e14194-s002.pdf]

Model 1

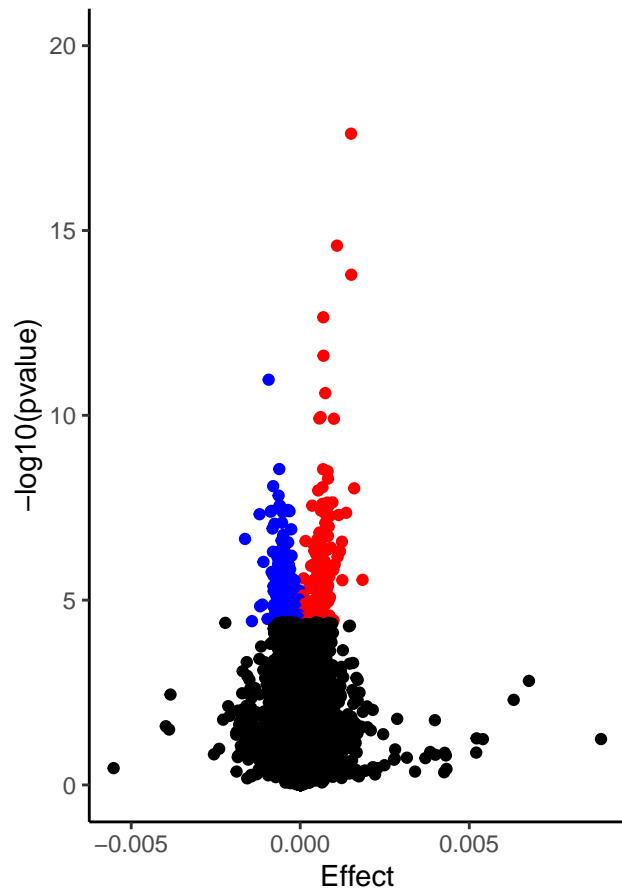

Model 2

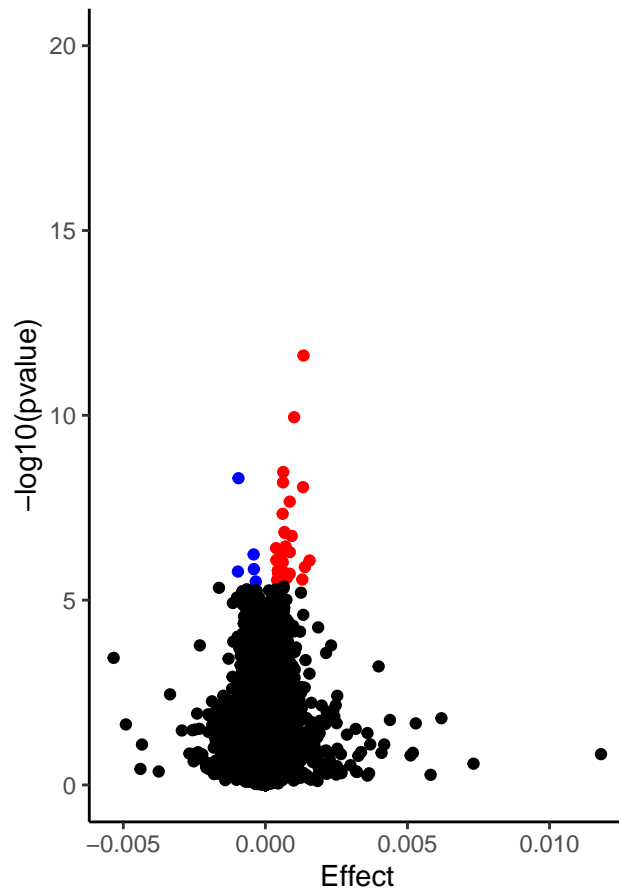

Model 3

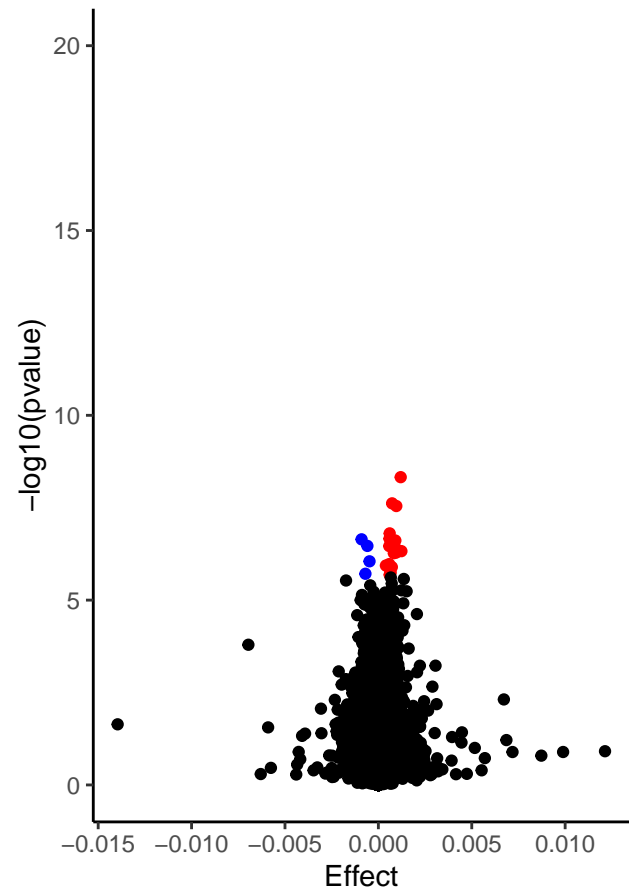

Supplement: Supplementary file 2 — Figure S2. [file ACEL-23-e14194-s001.pdf]

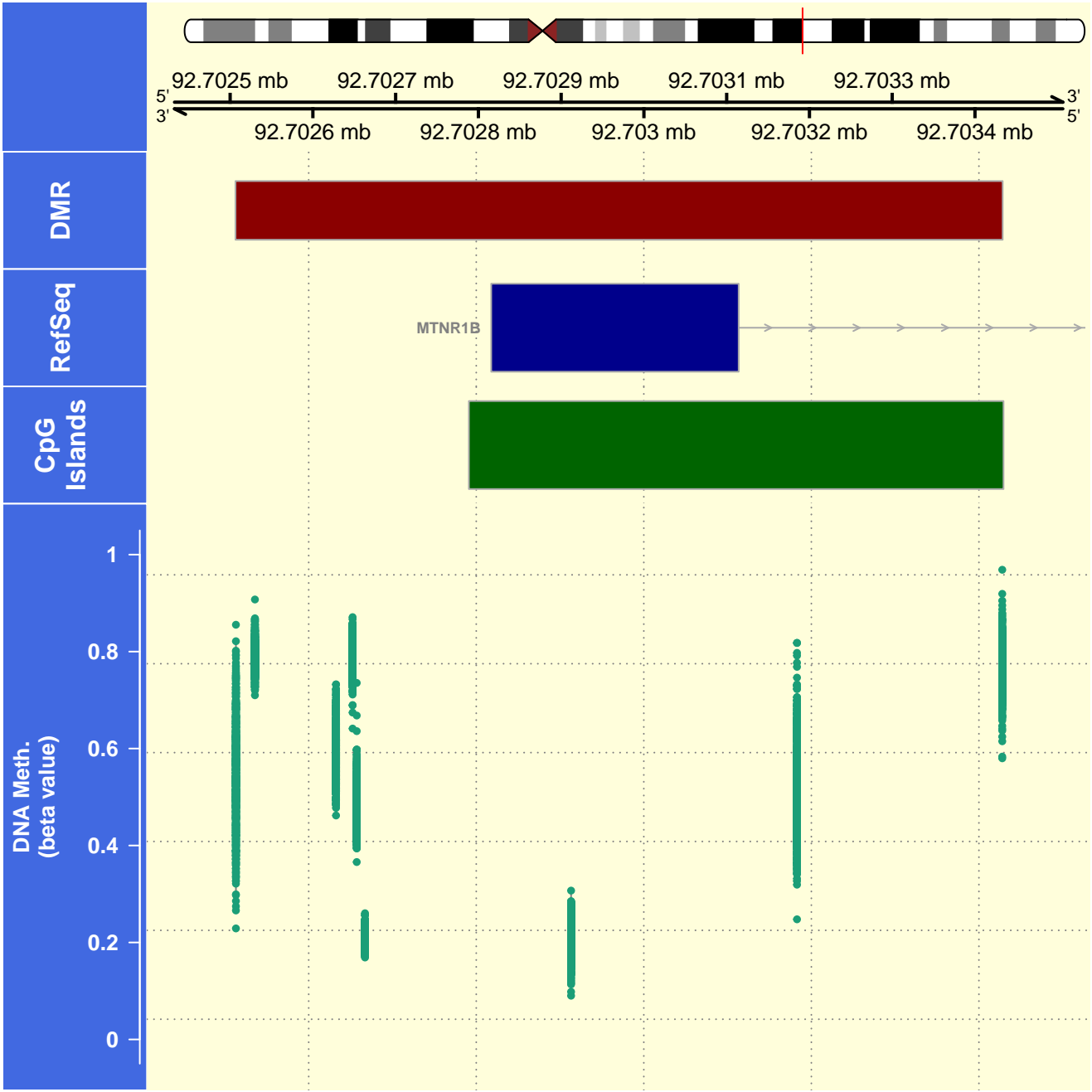

Supplement: Supplementary file 3 — Figure S3. [file ACEL-23-e14194-s004.pdf]
