# Supplementary material for: Maternal age is related to offspring DNA methylation: A meta‐analysis of results from the PACE consortium
Source: Aging Cell. 2024 May 29;23(8):e14194. doi: 10.1111/acel.14194 (PMC11320347; doi:10.1111/acel.14194)
Supplement: Supplementary file 4 — Figure S4. [file ACEL-23-e14194-s003.pdf]

DMPs analyzed across samples for erc2−DHS Maternal_age_newborn_main_model_EWAS_with_217cpgs

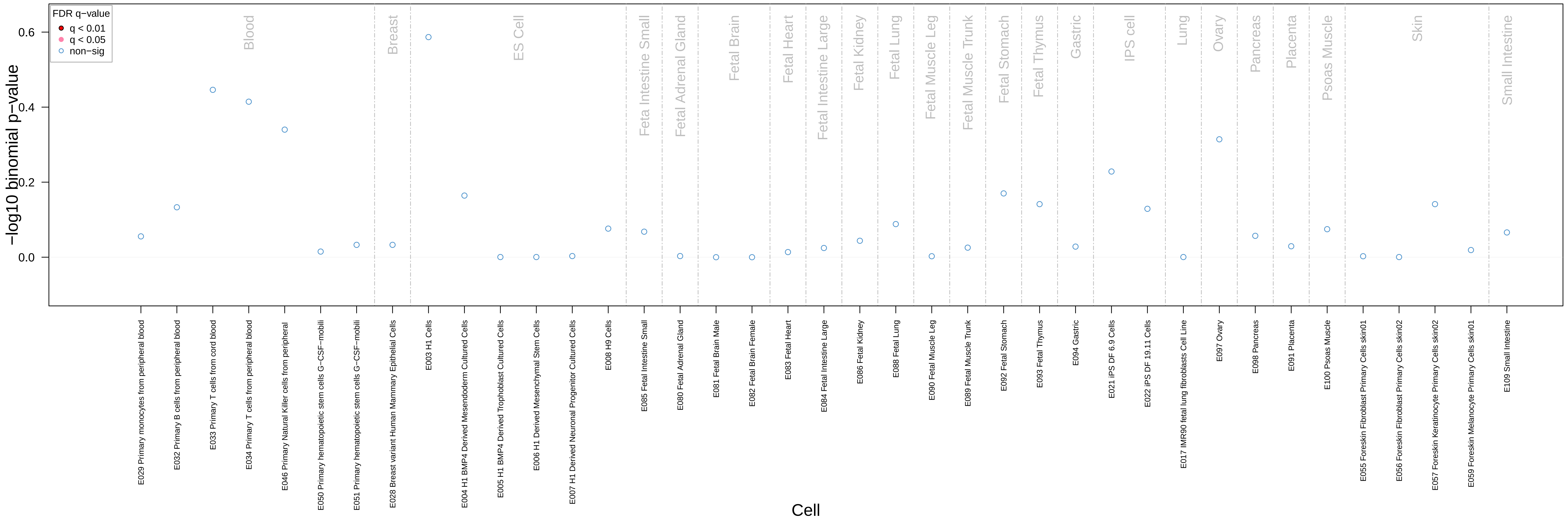

DMPs analyzed across samples for erc2−H3−all Maternal_age_newborn_main_model_EWAS_with_217cpgs

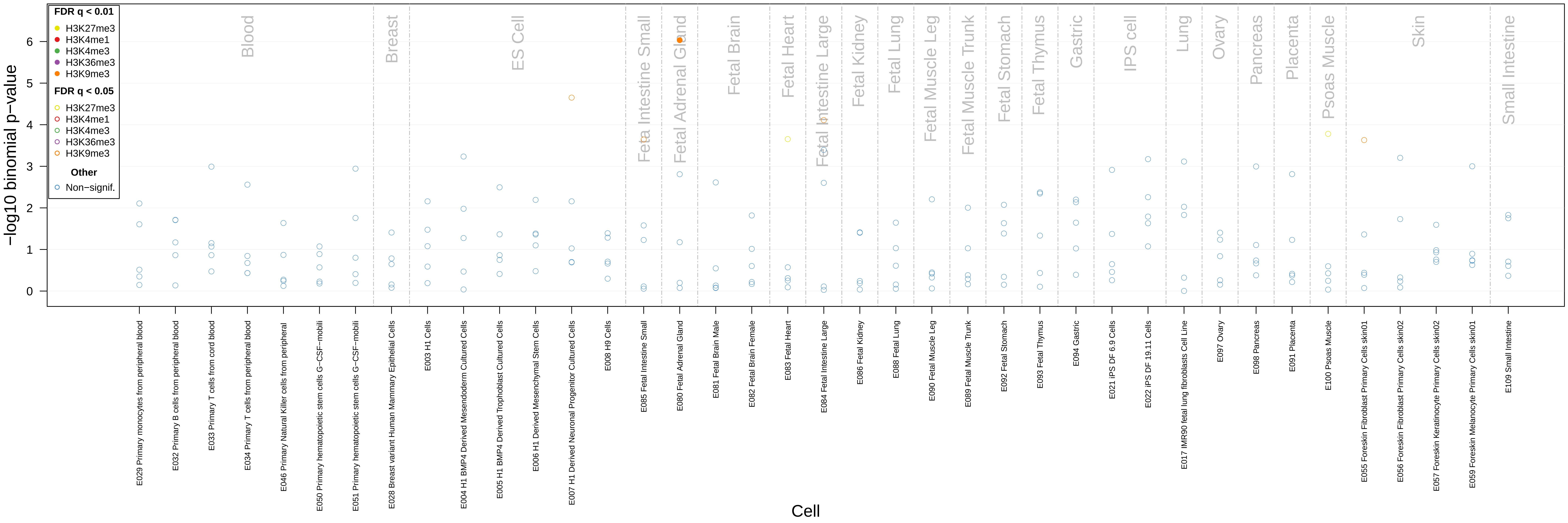

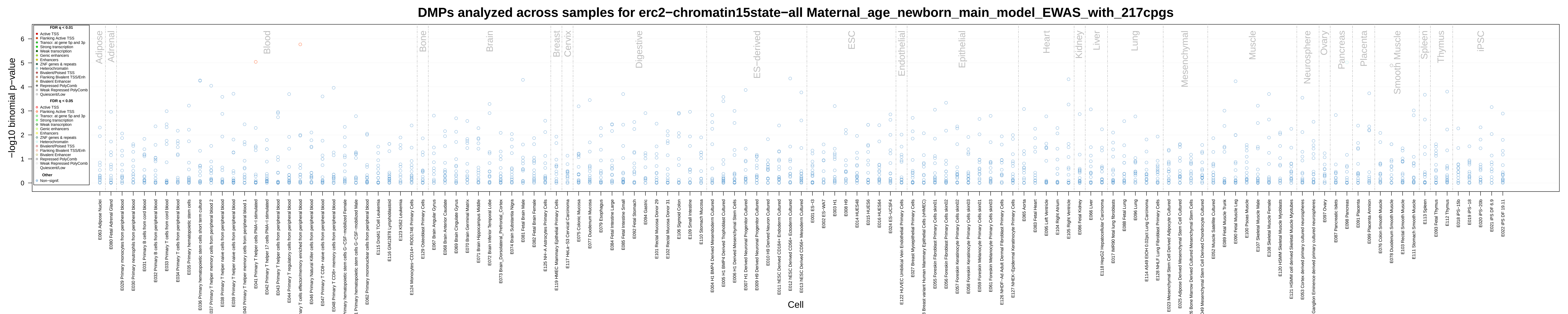
DMPs analyzed across samples for erc2−chromatin15state−all Maternal_age_newborn_main_model_EWAS_with_217cpgs
−log10 binomial p−value
Cell
FDR q < 0.01
FDR q < 0.05
Other
Non−signif.
Active TSS
Flanking Active TSS
Transcr. at gene 5p and 3p
Strong transcription
Weak transcription
Genic enhancers
Enhancers
ZNF genes & repeats
Heterochromatin
Bivalent/Poised TSS
Flanking Bivalent TSS/Enh
Bivalent Enhancer
Repressed PolyComb
Weak Repressed PolyComb
Quiescent/Low
Adipose
Adrenal
Blood
Bone
Brain
Breast
Cervix
Digestive
ES−deriv
ESC
Endothelial
Epithelial
Heart
Kidney
Liver
Lung
Mesenchymal
Muscle
Neurosphere
Ovary
Pancreas
Placenta
Smooth Muscle
Spleen
Thymus
iPSC
