# Supplementary material for: Maternal age is related to offspring DNA methylation: A meta‐analysis of results from the PACE consortium
Source: Aging Cell. 2024 May 29;23(8):e14194. doi: 10.1111/acel.14194 (PMC11320347; doi:10.1111/acel.14194)
Supplement: Supplementary file 6 — Data S1. [file ACEL-23-e14194-s005.docx]

**Supplemental information**

For manuscript: Maternal age is related to offspring DNA methylation: a meta-analysis of results from the PACE consortium

**Cohort Specific Methods, Funding, Disclosures and Acknowledgements**

**Supplemental background on *SHANK1*, *C2orf81*, *BOLA2B/YPEL3***

Avon Longitudinal Study of Parents and Children (ALSPAC)

*Cohort description*. Pregnant women resident in Avon in the UK with expected dates of delivery between 1st April 1991 and 31st December 1992 were invited to take part in the study^1,2^. The initial number of pregnancies enrolled was 14,541. Of the initial pregnancies, there was a total of 14,676 foetuses, resulting in 14,062 live births and 13,988 children who were alive at 1 year of age. Of the original 14,541 initial pregnancies, 338 were from a mother who had already enrolled with a previous pregnancy, meaning 14,203 unique mothers were initially enrolled in the study.

Phenotype data were collected using questionnaires. Data on maternal measures (e.g., age at delivery, ethnicity, education, smoking, parity) were collected from mothers via questionnaires administered during pregnancy and up to the time at which children were 8 weeks old. Partners were invited to complete questionnaires by the mothers at the start of the study but they were not formally enrolled at that time. In total, 8,350 partners provided their date of birth, with which ALSPAC researchers calculated their age when their baby was born. Biological samples and clinical measures were also obtained at several timepoints, including at birth. Please note that the study website contains details of all the data available through a fully searchable data dictionary and variable search tool: <http://www.bristol.ac.uk/alspac/researchers/our-data/>.

*Blood and methylation data*. In this study, we considered a subsample of 1018 children from the ALSPAC cohort who had DNA methylation data generated from cord blood (either white blood cells or blood spots) as part of the Accessible Resource for Integrative Epigenomic Studies (ARIES) sub-study^3^. Cord blood and peripheral blood samples (whole blood, buffy coats, white blood cells or blood spots) were collected according to standard procedures. Here, we only used cord samples (either white blood cells or blood spots) taken at birth.

Samples were profiled using the Illumina Infinium HumanMethylation450 BeadChip array. Functional normalisation was conducted using the *meffil* R package using standard parameters.^4^ Duplicates and samples showing evidence of population stratification based on ALSPAC genetic data were removed. After processing, 887 (97%) samples with cord blood methylation data and 482,855 probes remained.

We further excluded samples with “non-white" or missing ethnicity, defined as mothers or fathers with ethnicity different to "White" or missing. We identified <5 sets of twins in the dataset and randomly kept one twin per pair. Overall, 852 samples remained for the analysis. Because 27% of the samples that are not missing maternal age are missing paternal age, we ran model 2A, excluding samples that are missing paternal age. We recoded body mass index (BMI) outliers, setting BMI values to NA rather than removing the samples from the dataset because BMI is only needed for models 3 and 8. BMI outliers were defined as values above or below 5 standard deviations.

*Exposure and covariate information.* Phenotype data was collected using questionnaires. These were administered at several time points. Details can be found online^5^. Please note that the study website contains details of all the data available through a fully searchable data dictionary and variable search tool: <http://www.bristol.ac.uk/alspac/researchers/our-data/>.

Self-reported maternal and paternal ethnicity, if missing, was imputed using the ethnicity reported by the partner. Pre-pregnancy BMI was calculated from self-reported maternal height and pre-pregnancy weight. Paternal BMI was also based on self-reported height and weight. Self-reported parental education was grouped into 4 categories corresponding to 10, 13, 15, and 19+ years of education. Parity was derived from question on total live and stillbirths, subtracting stillbirths and setting negative values to missing. From repeated self-reports of smoking information throughout pregnancy, mothers were categorized into two groups: those that never smoked during pregnancy and those that did at least once in any of the three points solicited. Paternal smoking was dichotomized into never smokers or those who stopped before pregnancy and those who smoked at preconception and early pregnancy.

*Technical covariates*. We calculated 20 surrogate variables for each model and included them as covariates to account for batch effects and other unwanted sources of variation. Gervin (2016^6^) cord blood cell type reference dataset was used to estimate cell type proportions implemented in *meffil^4^,* generating "CD14", "Bcell", "CD4T", "CD8T", "NK", "Gran". *Meffil* normalizes each sample individually to the cell type reference dataset to avoid having cell count estimates depend on the other samples being included in the normalization.

Ethical approval: Ethical approval for the study was obtained from the ALSPAC Ethics and Law Committee and the Local Research Ethics Committees. Consent for biological samples has been collected in accordance with the Human Tissue Act (2004). Informed consent for the use of data collected via questionnaires and clinics was obtained from participants following the recommendations of the ALSPAC Ethics and Law Committee at the time.

Funding: HRE works in the Medical Research Council Integrative Epidemiology Unit at the University of Bristol, which is supported by the Medical Research Council and the University of Bristol (MC_UU_00011/5). RCR was supported by a Cancer Research UK grant (C18281/A29019). The UK Medical Research Council and Wellcome (Grant ref: 217065/Z/19/Z) and the University of Bristol provide core support for ALSPAC. This publication is the work of the authors and EY will serve as guarantors for the contents of this paper. This research was funded in whole, or in part, by the Wellcome Trust (224982/Z/22/Z). For the purpose of Open Access, the author has applied a CC BY public copyright licence to any Author Accepted Manuscript version arising from this submission. A comprehensive list of grants funding is available on the ALSPAC website (http://www.bristol.ac.uk/alspac/external/documents/grant-acknowledgements.pdf); This research was specifically funded by grants from the BBSRC (BBI025751/1; BB/I025263/1), IEU (MC_UU_12013/1; MC_UU_12013/2; MC_UU_12013/8), National Institute of Child and Human Development (R01HD068437), NIH (5RO1AI121226-02), and CONTAMED EU (212502). ALSPAC data were analysed under research proposal B4062.

Conflicts of Interest: None

Data Availability: The ALSPAC study website contains details of all the data available through a fully searchable data dictionary and variable search tool: <http://www.bristol.ac.uk/alspac/researchers/our-data/>.

Acknowledgements: We are extremely grateful to all the families who took part in this study, the midwives for their help in recruiting them, and the whole ALSPAC team, which includes interviewers, computer and laboratory technicians, clerical workers, research scientists, volunteers, managers, receptionists and nurses.

**Center for the Health Assessment of Mothers and Children of Salinas (CHAMACOS)**

Between October 1999 and October 2000, the Center for the Health Assessment of Mothers and Children of Salinas (CHAMACOS) study recruited 601 pregnant women from farmworker communities of Salinas Valley in California. At enrollment, women were ≤20 weeks gestation, English- or Spanish-speaking, Medicare eligible, planning to deliver at the county hospital, and attending prenatal care visits at one of the six local community clinics or hospitals. Of 601 initial enrollees of the cohort, 526 were followed to delivery of live, singleton newborns in 2000–2001.^7^ For this study, we included a subset of children with DNAm measured from cord blood specimens (N = 381). Nine of these participants were excluded due to missing reported sex or mismatch between reported sex and predicted sex derived from X chromosome intensities. These samples were deemed of low quality based on array intensity. Based on this exclusion criterion, the final sample included 372 children. There were 360 (96.8%) mothers and 363 (97.6%) fathers of the 372 children who identified as Latino. Due to small sample size in other race/ethnicity groups, analysis was limited to N = 357 children with both parents identifying as Latino.

Cord blood specimens were collected at the time of delivery, and DNA was extracted from the banked non-heparinized umbilical cord using QIAamp DNA Blood Maxi Kits (Qiagen, Valencia, CA) according to the manufacturer’s protocol with some modifications, as previously described.^8^ DNA aliquots of 1 µg were bisulfite converted using Zymo Bisulfite Conversion Kits (Zymo Research, Orange, CA). After whole-genome amplification, enzymatic fragmentation, and purification, DNA was applied to the Illumina Infinium HumanMethylation450 BeadChips (Illumina, San Diego, CA) according to the Illumina methylation protocol.^9,10^ Genome-wide DNAm levels were measured at 485,512 CpG sites on the 450K array. Quality control steps included the use of repeats and randomization of samples across chips and plates.^11^ Data processing was completed using the *minfi* R package^12^ and involved removal of poor-quality samples, filtering of probes containing common SNPs, cross-reactive probes, or low-quality probes (detection p-values >0.01), background correction, dye bias adjustment using the ‘noob’ method, and quantile normalization, as described elsewhere.^12,13^ Proportions of seven cell types (CD8T cells, CD4T cells, natural killer cells, B cells, monocytes, granulocytes, and nucleated red blood cells) were estimated from cord blood methylation profiles, using the method of Gervin et al.^14^

Data on maternal characteristics primarily came from medical records and maternal interviews conducted during pregnancy at around 13 and 26 weeks gestation and shortly after delivery. Clinical variables of interest were maternal age at delivery and pre-pregnancy body mass index (BMI). Pre-pregnancy weight was obtained from prenatal medical records. If pre-pregnancy weight was missing from medical records, weight at the first prenatal visit (≤13 weeks gestation) was used. Other covariates obtained from prenatal study interviews included maternal smoking during pregnancy (no, yes), baseline maternal education status (≤6th grade, 7–12th grade, ≥high school graduate), maternal parity. Mothers were asked about paternal race/ethnicity, age, smoking during pregnancy, and education at baseline.

Ethical Approval: The University of California, Berkeley Committee for the Protection of Human Subjects approved all study activities. Written, informed consent was obtained for all participating women, child verbal assent was obtained starting at age 7 years, child written assent was obtained starting at age 12 years, and child written consent was obtained at age 18 years.

Funding: This work was supported by the National Institutes of Health (R03AG067064; R01ES031259; R01ES026994; 1R01ES021369; P01ES009605; R01ES017054; R01DA035300; R24ES028529; R01MD0165595; and U24ES028529) and by the Environmental Protection Agency (R82670901; RD83171001; and RD83451301).

Conflicts of Interest: The authors declare that they have no conflicts of interest.

Data Availability: Investigators interested in using the CHAMACOS data can submit a data use application to the Berkeley CERCH.

**Children’s Health Study (CHS)**

The Children’s Health Study (CHS) is a population-based prospective cohort study from age 5 onwards in Southern California.^15^ A total of 5341 children were recruited, all of whom were born between 1995 and 1997. DNA methylation was measured in 273 Hispanic and non-Hispanic children, using DNA extracted from newborn dried bloodspots archived by the state of California. Twenty-seven children whose race were not White were excluded from this analysis due to small sample size. Additionally, 4 twins were excluded, followed by 11 children missing information on paternal age, and 6 children missing information on parental smoking. Eventually, 225 children were included in the analyses.

Laboratory personnel were blinded to study subject information. DNA was extracted from whole blood cells using the QiaAmp DNA blood kit (Qiagen Inc, Valencia, CA) and stored at -80 degrees Celsius. Genomic DNA was treated with bisulfite using the EZ-96 DNA Methylation Kit™ (Zymo Research, Irvine, CA, USA). The results of the Infinium HumanMethylation450 BeadChip were compiled for each locus as previously described and were reported as beta (β) values.^16^ Quality control of analyzed samples was performed using standardized criteria. Illumina Infinium 450K data were processed using the minfi package (version 1.16.0) in R^12^, after which a normal-exponential dye bias correction was applied to the raw intensities at the array level to reduce background noise.^17^ We then normalized each sample’s methylation values to have the same quantiles to address sample to sample variability.^18^ CpG loci on the array were removed from analyses if they contained SNPs, deletions, repeats, or if they have more than 10% missing values, leaving a total of 384,310 probes for the current analysis. Seven cell sub-populations were estimated using the combined cord blood cell reference with the EstimateCellCounts2 function.^14^ Estimated cord blood cell subpopulations (CD8+ T-lymphocytes, CD4+ T-lymphocytes, natural killer cells, B-lymphocytes, monocytes, granulocytes and nucleated red blood cells) were subsequently included as linear predictors in regression models.

Information on parental age (continuous, year), race (White, Black, Asian, Other), education (highest grade completed), newborn’s sex (female, male), parity (parous/not), gestational diabetes (yes/no), gestational age at birth (continuous, week), and birth weight (continuous, gram) was extracted from birth records. Parental education was recoded as continuous variables (year) in accordance with the International Standard Classification of Education 1997 classification equivalent to US years of schooling in the study. Information on parental smoking during pregnancy was retrospectively collected from the baseline questionnaire when children were 5 years old.

Ethical Approval: The study protocol was approved by the University of Southern California Institutional Review Board and informed, written consent and assent were provided by the parents and children respectively.

Funding: The CHS was supported by the following NIH grants: K01ES017801, R01ES022216, P30ES007048.

Conflicts of Interest: None

Data Availability: Data are available upon reasonable request to Dr. Breton (breton@usc.edu).

Acknowledgements: We would like to express our sincere gratitude to Martin Kharrazi, Steve Graham, and Robin Cooley at the California Biobank Program and Genetic Disease Screening Program within the California Department of Public Health for their assistance and advice regarding newborn bloodspots. The biospecimens and/or data used in this study were obtained from the California Biobank Program, (SIS request number(s) 479)” Section 6555(b), 17 CCR. The California Department of Public Health is not responsible for the results or conclusions drawn by the authors of this publication.

**Drakenstein Child Health Study (DCHS)**

Data were collected through the Drakenstein Child Health Study (DCHS), a population-based birth cohort in the Western Cape of South Africa. DNA was isolated from cord blood samples that were collected at the time of delivery.^19^ DNA methylation was assessed with the Illumina Infinium HumanMethylation450 (n=156) and the MethylationEPIC BeadChips (n=160). Pre-processing and statistics were done using R 3.5.1. Raw iDat files were imported to RStudio where intensity values were converted into beta values. The 450K and EPIC datasets were then combined using the minfi package^12^ resulting in 316 samples and 453,093 probes. Background subtraction, color correction and normalization were performed using the preprocess Funnorm function.^20^ Samples containing maternal blood contamination (n = 33) were removed.^19^ After removing pre-processing technical replicates (n = 7) and sex-mismatch (n = 3), a total of 273 samples remained.

Probes with NAs in ≥ 1% of samples or had a detection p value ≥ 1x10-16 in ≥ 1% of samples were removed (n = 10,868). Probes which bind to the sex chromosomes were removed due to the distribution differences observed (n = 9,896). Probes whose sequence contains a SNP either at the CpG site being measured or at the site of the single base pair extension with a minor allele frequency ≥ 1% were removed (n = 13,598).^21,22^ Autosomal probes which were in silico predicted to non-specifically bind to sex chromosomes in the genome were also removed (n = 9,698) leaving a total of 409,033 probes remaining for downstream analysis.^21,22^

Batch effects were removed using ComBat from the R package sva.^23^ Cord blood cell type composition was estimated using a cord blood reference data set^14^ and the IDOL algorithm and probe selection.^24^

Exposure and covariate information

After providing consent, participants completed a battery of self-report and clinician-administered measures throughout antenatal and postnatal study visits^9^. Maternal body mass index (BMI) and previous pregnancies were recorded at enrollment, and sociodemographic information was collected with an interviewer-administered questionnaire adapted from the South African Stress and Health Study (SASH) and included an assessment of highest level of education and race^10^. Prenatal smoking was measured using urine cotinine levels, taken within 4 weeks of enrollment, and measured with the IMMULITE 1000 Nicotine Metabolite Kit.^11^ Maternal age at birth was recorded 28-32 weeks gestation and used to calculate advanced maternal age (≥35 years at delivery). Offspring information was recorded from medical records and interviews during postnatal visits.

Ethical Approval: All Drakenstein study participants gave informed consent to participate, and the study was approved by University of Cape Town IRB.

Funding: The Drakenstein Child Health Study was funded by the Bill & Melinda Gates Foundation (OPP 1017641, OPP1017579), Medical Research Council South Africa, and the National Research Foundation South Africa. Additional support for the DNA methylation work was by the *Eunice Kennedy Shriver* National Institute of Child Health and Human Development of the National Institutes of Health (NICHD) under Award Number R21HD085849, and the Fogarty International Center (FIC). AH and EC were supported by the HERCULES Center (NIEHS P30ES019776). DJS and HJZ are supported by the South African Medical Research Council (SAMRC).

Conflicts of interest: The authors declare they have nothing to disclose.

Data Availability: NA

Acknowledgements: The authors thank the study and clinical staff at Paarl Hospital, Mbekweni and TC Newman clinics, as well as the CEO of Paarl Hospital, and the Western Cape Health Department for their support of the study. The authors thank the families and children who participated in this study. The authors also thank Dr. Michael S. Kobor and his team at the University of British Columbia for the generation, pre-processing and quality control of the DNA methylation data (data generation: Julia L MacIsaac, David TS Lin, Katia E Ramadori; pre-processing/quality control: Nicole Gladish).

**Effects of Aspirin in Gestation and Reproduction (EAGeR) trial**

The Effects of Aspirin in Gestation and Reproduction (EAGeR) trial (2007-2011) was a multi-site clinical trial which randomized 1,228 women with a history of pregnancy loss to low-dose aspirin and folic acid or folic acid alone prior to conception.^25,26^ Women aged 18-40 years old, with a history of 1 or 2 prior pregnancy losses, no history of infertility, actively trying to conceive, and with regular menstrual cycles during the past year, were eligible for the trial. Exclusion criteria included any prior diagnosis of infertility or sub-fertility. Women were followed for up to six menstrual cycles while attempting pregnancy and then monthly throughout pregnancy for women who conceived (n=595 delivered a live birth). At the Utah study site, cord blood was collected beginning in 2009 and was obtained for over 90% of deliveries (n=428), of whom 397 singletons had sufficient DNA available.^27^ Of note, randomization to low-dose aspirin had no impact on DNAm in cord blood and so no further exclusions or adjustments were made for randomization in the current analysis.^27^ Maternal age, parity, and gestational diabetes were derived from medical records. Additional information was self-reported on questionnaires including smoking (yes/no), education (years), and race/ethnicity. Height and weight were clinically measured approximately 2 months prior to pregnancy from which body mass index was derived.^27^ No information was collected on paternal age.

DNA extracted from cord blood buffy coat was bisulfite-converted using the EZ MethylationTM kit (Zymo, Irvine, CA). Methylation was measured using the MethylationEPIC 850K BeadChip microarray (San Diego, CA). Data were processed using the minfi package^28^ including background and dye-bias corrections. Quantile normalization was used to normalize beta values.^18^ Samples were cleaned of sex mismatch (n=5) by examining principal components. We extracted the detection p-value for each methylation measure (per site per sample) and filtered data which failed detection (n=1, p>0.01). After those QC exclusions, 391 singletons remained. Beta values were dropped if either detection p-value > 0.01 or bead counts < 3. We removed samples and CpG sites with low passing rate (<97%) based on detection p-value and bead counts. After probe removal, 833,937 CpG probes remained. A total of 358 (92% of 391) mother-child pairs were included in the current analysis after exclusions for non-White race due to few samples (n=12) and missing covariate information.

Ethical approval: IRB approval was attained prior to enrollment (Salt Lake City, Utah IRB #1002521). All participants provided written informed consent.

Funding: This work was supported by the Intramural Research Program of the *Eunice Kennedy Shriver* National Institute of Child Health and Human Development (National Institutes of Health, Bethesda, MD, USA) under contract numbers HHSN267200603423, HHSN267200603424, HHSN267200603426, and HHSN275201300023I-HHSN2750008.

Conflicts of interest: None

Data availability: The data for the current analysis is available from the corresponding author (E.Y.) on reasonable request pending application and approval.

Acknowledgements: This work utilized the computational resources of the NIH High Performance Computing Biowulf cluster (http://hpc.nih.gov).

**Early Autism Risk Longitudinal Investigation (EARLI)**

The Early Autism Risk Longitudinal Investigation (EARLI) is an enriched risk prospective pregnancy cohort to study autism etiology.^29^ This longitudinal study recruited mothers of confirmed ASD children who were early in a subsequent pregnancy or were trying to become pregnant. There were 232 mothers with a subsequent sibling born through this study. All children were born between November 2009 and March 2012. Cord blood was collected for 175 births. DNA was extracted using the DNA Midi kit (Qiagen, Valencia, CA) and samples were bisulfite treated and cleaned using the EZ DNA methylation gold kit (Zymo Research, Irvine, CA). DNA was plated randomly and assayed on the Infinium HumanMethylation450 BeadChip (Illumina, San Diego, CA) at the Johns Hopkins SNP Center, a shared lab and informatics operation with the Center for Inherited Disease Research (Johns Hopkins University). Methylation control gradients and between-plate repeated tissue controls were used.

Methylation beta values were processed with noob normalization, as well as 2% winsorized. Probes with failed detection P-value (>0.01) in >10% of samples were removed (n=661), leaving a total of 484,851 probes. Samples with discordant methylation predicted sex and observed sex were removed (n=3). There was a twin pair, one of which was randomly excluded from analysis (n=1).

Demographics, maternal behaviors, food frequency, medical history were all collected via questionnaire. Only a few mothers smoked during pregnancy, thus they (n=6) were excluded from analysis, and the smoking covariate was not included in models. Additionally, principal components from genotype data were included as covariates. Hybridization date, of which samples were spread across two, was used as a batch variable. Cell type estimation was based on the Gervin 2019 cord blood reference.^14^

Ethical statement: The EARLI study was reviewed and approved by Human Subjects Institutional Review Boards (IRBs) from each of the four study sites (Johns Hopkins University, Drexel University, University of California Davis, and Kaiser Permanente Northern California).

Funding: Funding for the EARLI study was provided by the National Institutes of Health (R01 ES016443, PI: Newschaffer; R24ES030893, PI: Fallin; R01ES025531, PI: Fallin) and Autism Speaks (003953 PI: Newschaffer). Mr. Dou and Dr. Bakulski were supported by grants from the National Institutes of Health (R01 ES025531, PI: Fallin; R01 MD013299). The content is solely the responsibility of the authors and does not necessarily represent the official views of the National Institutes of Health.

Conflicts of Interest: None.

Data Availability: Data are available through the National Database for Autism Research (NDAR) accession number for EARLI (1600).

**Generation R Study (GenR)**

The Generation R Study is a population-based prospective cohort study from fetal life onwards established in Rotterdam, the Netherlands.^30^ Pregnant women with an expected delivery date between April 2002 and January 2006 living in Rotterdam were invited to participate, and written informed consent was obtained from all participants. In 1396 of the 9901 live-born newborns we measured genome-wide DNA methylation in cord blood. This subgroup was selected from the total study population as a relatively homogeneous, European-ancestry subgroup. We only included children for whom the mother’s partner was also the biological father of the child, and 135 participants were excluded based on this criterion. A total of 13 mothers had two (non-twin) children and only one child per mother was kept; based on fewer missing covariate values (7 children) or at random for sibling pairs for whom covariates were complete (6 children). Additionally, 23 mother-child pairs were excluded from the analysis due to maternal self-reported non-European ethnicity. In the current analysis, we included mother–newborn pairs who had complete information on the exposure and covariate variables, a total of 1225 participants.

We used the salting-out method to extract DNA from cord blood samples. Five-hundred nanograms of DNA were bisulfite converted using the EZ-96 DNA Methylation kit (Shallow) (Zymo Research Corporation, Irvine, USA). Samples were processed with the Illumina Infinium HumanMethylation450 BeadChip (Illumina Inc., San Diego, USA). Quality control and normalization were performed using the CPACOR workflow.^31^ Probes with a detection p ≥ 1E−16 were set to missing. Intensity values were quantile normalized. We removed arrays with technical problems, a call rate ≤ 95%, or a mismatch between the expected sex of participant and sex determined by chromosome X and Y probe intensities. Probes on the sex chromosomes were removed before the analyses. The final number of probes was 458,563. We used untransformed beta-values as measures of DNA methylation.

Paternal and maternal age was self-reported and collected from questionnaires sent out during pregnancy. Maternal covariate information was obtained via maternal questionnaires sent out in each pregnancy trimester and height and weight were measured during the first visit in pregnancy at a dedicated research center. For maternal BMI calculation, we used weight measured at the research center if the first visit was before 15 weeks gestational age. If the first visit was after 15 weeks, pre-pregnancy self-reported weight was used. Highest level of completed education was categorized into two categories: low and medium education versus higher education (college and university degree), parity as nulliparous versus multiparous, maternal smoking during pregnancy was categorized into no smoking versus any pregnancy smoking. For paternal smoking in the months preceding pregnancy, we used the maternal report, as it was more complete and highly correlated with paternal self-report. Paternal smoking was categorized into no/yes. The remaining paternal covariates, height, weight, and education, were collected from the partner questionnaire sent during pregnancy. Batch effects were accounted for by adding sample plate number as a covariate. Cord blood cell type proportions were estimated a cord blood reference using the “FlowSorted.CordBlood.Combined.450 K” Bioconductor package.^14^ This reference set included: CD8+ T cells, CD4+ T cells, natural killer cells, B cells, monocytes, granulocytes, nucleated red blood cells.

Ethical Approval: The Generation R Study has been approved by the Medical Ethical Committee of Erasmus MC, University Medical Center Rotterdam.

Funding: The general design of the Generation R Study is made possible by financial support from Erasmus MC, University Medical Centre Rotterdam, Erasmus University Rotterdam, the Netherlands Organization for Health Research and Development (ZonMw), the Netherlands Organization for Scientific Research (NWO), the Ministry of Health, Welfare and Sport, and the Ministry of Youth and Families. The EWAS data was funded by a grant to VWVJ from the Netherlands Genomics Initiative (NGI)/Netherlands Organization for Scientific Research (NWO) Netherlands Consortium for Healthy Aging (NCHA; project number 050-060-810), by funds from the Genetic Laboratory of the Department of Internal Medicine, Erasmus MC, University Medical Centre Rotterdam (R01HD068437). The project was supported by funding from the European Union’s Horizon 2020 research and innovation program under grant agreements No 733206 (LifeCycle), 874739 (LongITools) and 874583 (ATHLETE), and from the European Joint Programming Initiative ‘A Healthy Diet for a Healthy Life’ (JPI HDHL, NutriPROGRAM project, ZonMw the Netherlands no. 529051022).

Conflicts of Interest: None

Data Availability: Data from this study are available upon reasonable request to the director of the Generation R Study ([generationr@erasmusmc.nl](mailto:generationr@erasmusmc.nl)), subject to local, national and European rules and regulations.

Acknowledgements: The Generation R Study is conducted by Erasmus MC in close collaboration with the School of Law and Faculty of Social Sciences of the Erasmus University Rotterdam, the Municipal Health Service Rotterdam area, Rotterdam, the Rotterdam Homecare Foundation, Rotterdam, and the Stichting Trombosedienst & Artsenlaboratorium Rijnmond (STAR-MDC), Rotterdam. We gratefully acknowledge the contribution of children and parents, general practitioners, hospitals, midwives, and pharmacies in Rotterdam. The generation and management of the Illumina 450K methylation array data (EWAS data) for the Generation R Study was executed by the Human Genotyping Facility of the Genetic Laboratory of the Department of Internal Medicine, Erasmus MC, and the Netherlands. We thank Mr Michael Verbiest, Ms Mila Jhamai, Ms Sarah Higgins, Mr Marijn Verkerk, and Dr Lisette Stolk for their help in creating the EWAS database. We thank Dr Alexander Teumer for his work on the quality control and normalization scripts.

**Gen3G**

The Genetics of Glucose regulation in Gestation and Growth (Gen3G) is a prospective observational cohort study aiming to increase our understanding of biological, environmental, and genetic determinants of glucose regulation during pregnancy and their impact on foetal development and was described in detail previously.^32^ In brief, we recruited a total of 1024 pregnant women aged ≥18 years old between January 2010 and June 2013 representing the general population of women in reproductive age receiving care at our institution. We excluded women if they had non-singleton pregnancy, known pre-pregnancy diabetes or overt diabetes diagnosed based on biochemical screening that we performed at first trimester.

Exposure and Covariates

Maternal age (continuous in years) is the age of the mother at delivery (calculated from date of birth and date of delivery – from medical records) rounded to the nearest integer. Parity was divided into 2 groups: 0=did not have a previous term or preterm delivery, 1=previously had ≥1 term or preterm delivery. Number of term and preterm deliveries was self-reported. Maternal smoking was divided into 2 groups: 0=No smoking at first trimester visit, 1=Smoking at first trimester visit (self-reported). Gen3G did not collect information on maternal education. Maternal BMI was based on first trimester measured height and weight (median gestational age: 9.6 weeks). Cell type distributions were estimated based on a cord blood reference.^14^

Data collection methods

Cord blood was collected at delivery. We purified DNA from 460 cord blood samples using the AllPrep DNA/RNA/Protein Mini Kit (QIAGEN). After bisulfite conversion, the Illumina Laboratory (San Diego, CA) performed epigenome-wide DNA methylation measurements using the Infinium MethylationEPIC BeadChip. We imported methylation data into R for preprocessing using minfi. We normalized our data as previously described.^33^ We applied functional normalization (FunNorm^20^) and Regression on Correlated Probes RCP^34^) to adjust for technical variability and probe type bias, respectively. Briefly, FunNorm removes technical variability using control probes from the array and RCP corrects type II probe distributions using the distribution of proximal type I probes to increase precision. Failed samples (n=3), sex and SNP mismatch (n=6) were removed. We also removed samples with potential contamination of mother’s blood (n=2) and we had no sample with too many failed probes (>5% with detection p-value>1.0E-5). We also removed probes with too may failed samples (>5% with detection p-value >0.05). We also excluded one sample with a chromosomal problem and samples of non-European ethnicity (n=8). We removed probes with too many failed samples (>5% with detection p-value >0.05). We used sample plate as a covariate in models to adjust for batch effect. We winsorized at 2% (1% each side) with the provided code. After QC and removal of non-European participants due to few numbers available, DNA methylation from 440 Gen3G participants, measured at 865,076 CpG probes remained for the current analysis.

Ethical Approval: The study protocol was approved by the Centre Hospitalier Universitaire de Sherbrooke (CHUS) ethic committee board and every participant gave written informed consent before enrollment in the study, in accordance with the Declaration of Helsinki.

Funding: Gen3G work presented in this study was supported by an American Diabetes Association Pathways Award #1-15-ACE-26 (MFH); Gen3G has also been supported by Fonds de recherche du Québec en santé #20697 (MFH); Canadian Institute of Health Research #MOP 115071 (MFH) and to LB #PJT-152989); and a Diabète Québec grant. LB is a senior research scholar from the FRQS.

Conflicts of Interest: None

Data Availability: Original Gen3G data is available to external investigators upon reasonable request to Gen3G Principal investigators (MFH and LB).

Acknowledgements: We thank participants of the Gen3G, as well as clinical research nurses and research assistants for recruiting women and obtaining consent. We also thank the CHUS biomedical laboratory for performing some assays.

**Human Early Life Exposome (HELIX)**

Human Early Life Exposome (HELIX) study represents a collaborative project across six established and ongoing longitudinal population-based birth cohort studies in Europe: the Born in Bradford (BiB) study in the UK, the Étude des Déterminants pré et postnatals du développement et de la santé de l’Enfant (EDEN) study in France, the INfancia y Medio Ambiente (INMA) cohort in Spain, the Kaunus cohort (KANC) in Lithuania, the Norwegian Mother, Father, and Child Cohort Study (MoBa) and the RHEA Mother Child Cohort study in Crete, Greece. The HELIX project aims to measure and describe multiple environmental exposures from the different exposome domains during early life (pregnancy and childhood) and associate these with omics markers and child health outcomes.^35,36^ The present study used data from a set of 1285 child participants (total recruited for the collaborative cohort was 1301 children) from all the cohorts and including all ancestries: BIB (N = 204), EDEN (N = 198), INMA (N = 223), KANC (N = 199), MoBa (N = 264), RHEA (N = 197).

Blood samples were collected at the 8-years follow-up visit at each cohort and processed and stored at -80°C, following the same harmonized protocol. DNA extraction from buffy coats from all the cohorts was done centrally in the same laboratory using the Chemagen kit (Perkin Elmer). DNA concentration was determined by NanoDrop spectrophotometer (Thermo Scientific) and with the Quant-iT PicoGreen dsDNA Assay Kit (Life Technologies). Methylation data was produced at the Spanish National Genotyping Center (CEGEN, Madrid) following the Illumina protocol for the Infinium HumanMethylation450 beadchip. Briefly, samples were randomized and then, 500 ng of DNA was bisulfite-converted using the EZ 96-DNA methylation kit following the manufacturer’s standard protocol, and DNA methylation was measured using the Illumina Infinium HumanMethylation 450 beadchip.

DNA methylation data were pre-processed using the minfi package.^12^ Probes not reaching a 98% call rate were excluded (detection p-value was defined at 10E-16 according to the guidelines^31^). Two samples were filtered due to overall low quality: one had a call rate <98% and the other did not pass QC parameters of the MethylAid package.^37^ Then, data were normalized with the functional normalization method, which also included Noob background subtraction and dye-bias correction.^17^ After that, several quality control checks were performed. First, we checked sex consistency using the shinyMethyl package^38^ and two samples were excluded. Genetic consistency of duplicates and samples from the same participant was checked with the 450K genotypes. In addition, genetic consistency was evaluated in those samples that had GWAS data and two of them were excluded. Principal component analysis showed no differential clusters, however a degree of grouping within the main cluster was observed for some biological variables (sex, cohort) and for some technical variables. ComBat was applied to correct the array technical batch effect.^39^ Duplicated samples and HapMap samples were removed as well as control probes, probes designed to detect SNPs and probes to measures methylation levels at non-CpG sites. The final dataset after quality control consisted of 1160 samples and 480,444 probes.

Maternal and paternal age, ethnicity, education, smoking behavior and parity (nulliparous, 1, or >= 2 previous children) were collected by questionnaire in each cohort. Child’s ethnicity was predicted from genome-wide genetic data using the program peddy. Maternal BMI was calculated from self-reported height and weight (EDEN, MoBa and KANC), self-reported height and measured weight (INMA), and measured height and self-reported weight (RHEA). Cell type composition was estimated using the reference panel described elsewhere.^40^ Cohort was included as a covariate in the models.

Ethical Approval: The six HELIX cohorts have the required permissions by national ethics committees for their cohort recruitment and follow-up visits and for secondary use of pre-existing samples and data. The work in HELIX was covered by new ethics approvals in each country. At enrolment in the HELIX project, families were asked to sign an informed consent form for the specific HELIX work including clinical examination and biospecimen collection and analysis. An Ethics Task Force was established to support the HELIX project on ethical issues, for advice on the project’s ethical compliance, identification and alerting to changes in legislation where applicable. Specific procedures are in place within HELIX to safeguard the privacy of study subjects and confidentiality of data.

Funding: The study has received funding from the European Community’s Seventh Framework Programme (FP7/2007-206) under grant agreement no 308333—the HELIX project. Born in Bradford (BiB) is supported by a Wellcome programme grant (WT223601/Z/21/Z: Age of Wonder) and an infrastructure grant (WT101597MA). INfancia y Medio Ambiente (INMA) data collections were supported by grants from the Instituto de Salud Carlos III, CIBERESP, the Generalitat de Catalunya-CIRIT. KANC was funded by the grant of the Lithuanian Agency for Science Innovation and Technology (6-04-2014_31V-66). The Norwegian Mother, Father and Child Cohort Study (MoBa) is supported by the Norwegian Ministry of Health and Care Services and the Ministry of Education and Research. The Rhea project was financially supported by European projects, and the Greek Ministry of Health (Program of Prevention of Obesity and Neurodevelopmental Disorders in Preschool Children, in Heraklion district, Crete, Greece: 2011–2014; 'Rhea Plus': Primary Prevention Program of Environmental Risk Factors for Reproductive Health, and Child Health: 2012–2015). The EDEN study was supported by Foundation for medical research (FRM), National Agency for Research (ANR), National Institute for Research in Public health (IRESP: TGIR cohorte santé 2008 program), French Ministry of Health (DGS), French Ministry of Research, INSERM Bone and Joint Diseases National Research (PRO-A), and Human Nutrition National Research Programs, Paris-Sud University, Nestlé, French National Institute for Population Health Surveillance (InVS), French National Institute for Health Education (INPES), the European Union FP7 programmes (FP7/2007–2013, HELIX, ESCAPE, ENRIECO, Medall projects), Diabetes National Research Program (through a collaboration with the French Association of Diabetic Patients (AFD)), French Agency for Environmental Health Safety (now ANSES), Mutuelle Générale de l’Education Nationale a complementary health insurance (MGEN), French national agency for food security, French-speaking association for the study of diabetes and metabolism (ALFEDIAM). The HELIX study has received funding from the European Joint Programming Initiative “A Healthy Diet for a Healthy Life” (JPI HDHL and Instituto de Salud Carlos III) under the grant agreement no AC18/00006 (NutriPROGRAM project). The work was also supported by MICINN (MTM2015-68140-R) and Centro Nacional de Genotipado-CEGEN-PRB2-ISCIII. We acknowledge support from the Spanish Ministry of Science and Innovation and the State Research Agency through the “Centro de Excelencia Severo Ochoa 2019-2023” Program (CEX2018-000806-S), and support from the Generalitat de Catalunya through the CERCA Program. LG received a fellowship from the Health Department of the Catalan Government (PERIS SLT017/20/000215).

Conflicts of Interest: The authors have no conflicts of interest to declare. All co-authors have seen and agree with the contents of the manuscript and there is no financial interest to report.

Data Availability: The raw data supporting the current study are available from the corresponding author on request subject to ethical and legislative review. The “HELIX Data External Data Request Procedures” are available with the data inventory in this website: <http://www.projecthelix.eu/data-inventory>. The document describes who can apply to the data and how, the timings for approval and the conditions to data access and publication.

Acknowledgements: We would like to thank all the children of the HELIX cohorts and their families for their generous contribution.

**INfancia y Medio Ambiente (INMA)**

The INfancia y Medio Ambiente (INMA) Project is a population-based mother–child cohort study in Spain (Guxens et al., 2012). The current study is based on the INMA Sabadell subcohort that includes 777 children recruited between 2004 and 2007 in the Sabadell city. In particular, we selected 385 children of European ancestry with information on parental age, cord blood DNA methylation and covariates. Study website: http:/www.proyectoinma.org/.

Methylation data acquisition:

Cord blood samples were collected at birth at the delivery hospital and were immediately processed and frozen at -80ºC. Cord blood DNA was extracted using the Chemagen kit (Perkin Elmer). DNA concentration was determined by NanoDrop spectrophotometer (Thermo Scientific) and with the Quant-iT PicoGreen dsDNA Assay Kit (Life Technologies). Methylation data was produced in two different laboratories as part of two different projects: in the Genome Analysis Facility of the University Medical Center Groningen (UMCG) in Holland, and in the Bellvitge Biomedical Research Institute (IDIBELL, Barcelona). Both laboratories used the recommended Illumina protocol for the Infinium HumanMethylation450 beadchip. Briefly, 500 ng of DNA was bisulfite-converted using the EZ 96-DNA methylation kit following the manufacturer’s standard protocol, and DNA methylation measured using the Illumina Infinium HumanMethylation450 beadchip.

Methylation data QC:

DNA methylation data were quality controlled and preprocessed using the minfi package.^12^ A series of steps were completed for quality control and data analysis. The first step was low quality sample removal. First, 2 samples with bad overall quality or with low detection p-value according to the output of the MethylAid package were removed.^37^ Then, we removed 3 samples whose sex was wrongly predicted using shinyMethyl.^38^ Following guidelines of Lehne’s work^31^, we increased the stringency of the detection p-value threshold to 10E-16 and we filtered 18 samples with a call rate lower than 98%. Data was normalized with the functional normalization method. Correlation between SNPs in replicated samples was checked and probes not measuring SNPs were discarded. 7136 probes with a call rate lower than 95% were also removed. Probes in sexual chromosomes, crosshibridizing or containing SNPs were flagged but not removed at this point. ComBat was applied to remove laboratory batch effect.^39^ Finally, duplicated samples were removed. The final dataset after quality control consisted of 385 cord blood samples from European-Caucasian ancestry (476,946 probes).

Exposure and covariate information:

Maternal age, ethnicity, education, pre-pregnancy weight, smoking behavior and parity (defined as the number of previous births before current pregnancy, including live births and stillborn ≥22 weeks) were collected by questionnaire at enrolment during week 12 of pregnancy. Paternal age, ethnicity, education, weight, height and smoking behavior were also collected by questionnaire in subsequent follow-up visits. BMI was calculated from measured height and self-reported pre-pregnancy weight at enrolment (reported pre-pregnancy weight was highly correlated with measured weight at 12 weeks of pregnancy: r= 0.96; P < 0.0001). Cell type composition was estimated using the reference panel for cord blood described elsewhere.^14^

Ethical Approval: The INMA study was approved by the Ethics Committee of the reference hospital (CEIm – Parc de Salut Mar), and all participants gave their written informed consent.

Funding: This study was funded by grants from Instituto de Salud Carlos III (Red INMA G03/176; CB06/02/0041; PI041436; PI081151 incl. FEDER funds), Generalitat de Catalunya-CIRIT 1999SGR 00241, Fundació La marató de TV3 (090430), EU Commission (261357-MeDALL: Mechanisms of the Development of ALLergy), and European Research Council (268479-BREATHE: BRain dEvelopment and Air polluTion ultrafine particles in scHool childrEn). We acknowledge support from the Spanish Ministry of Science and Innovation and the State Research Agency through the “Centro de Excelencia Severo Ochoa 2019-2023” Program (CEX2018-000806-S), and support from the Generalitat de Catalunya through the CERCA Program. LG received a fellowship from the Health Department of the Catalan Government (PERIS SLT017/20/000215).

Conflicts of Interest: The authors have no conflicts of interest to declare. All co-authors have seen and agree with the contents of the manuscript and there is no financial interest to report.

Data Availability: INMA databases are stored in ISGlobal (PRBB) servers and protected under internal cybersecurity regulations. Data are available upon request under signature of a data transfer agreement.

Acknowledgements: We particularly thank all the INMA participants for their generous contributions.

**Markers of Autism Risk Learning Early Signs (MARBLES)**

Markers of Autism Risk Learning Early Signs (MARBLES) is an enriched risk prospective pregnancy cohort to study autism etiology.^41^ This ongoing longitudinal study recruited mothers of confirmed ASD children who were in a subsequent pregnancy or were trying to become pregnant. At the time of this analyses, there were 389 enrolled mothers that gave birth to 425 subsequent siblings between December 1, 2006 and July 1, 2016. At the delivery hospital, cord blood samples were collected and immediately processed and frozen. Cord blood samples were stored at -80 degrees Celsius in the UC Davis repository. DNA methylation assays were run on 247 cord blood samples. DNA was plated randomly and assayed on the Infinium HumanMethylationEPIC BeadChip (Illumina, San Diego, CA) at the Johns Hopkins SNP Center, a shared lab and informatics operation with the Center for Inherited Disease Research (Johns Hopkins University). DNA methylation control gradients and between-plate repeated tissue controls were used.

Methylation beta values were processed with noob normalization, as well as 2% windorized. Probes were dropped (n=4,633) if they had detection-p (p>0.01) failure in greater than 5% of samples. Cross reactive probes (n=42,967) were also dropped^21^, leaving a total of 818,259 probes. Samples that had mismatched predicted sex were dropped (n=3).

Demographics, maternal behaviors, food frequency, medical history were all collected via questionnaire. Only a few mothers smoked during pregnancy, thus they (n=10) were excluded from analysis, and the smoking covariate was not included in models. Cell type estimation was based on the Gervin 2019 cord blood reference.^14^ Additionally, principal components from genotype data were included as covariates. Samples that did not have accompanying genotype data (n=6) were not included in this analysis. Sample plate, of which samples were spread across three, was used as a batch variable.

Ethical Approval: The MARBLES protocol was reviewed and approved by the Human Subjects Institutional Review Board (IRB) from University of California Davis.

Funding: The MARBLES study and this work has been supported by a grant from the Allen Foundation, pilot funding from the MIND Institute, EPA STAR grant #RD-83329201, and NIH grants: R01ES025574, R01ES029213, R24ES028533, R01ES028089, R01ES020392, P01ES011269, and K12HD051958. These supporting organizations had no role in the design and conduct of the work; collection, management, analysis, and interpretation of the data; preparation, review, or approval of the manuscript; and decision to submit the manuscript for publication. The findings and conclusions in this report are those of the authors and do not necessarily represent the official position of the National Institutes of Health or EPA.

Conflicts of Interest: None.

Data Availability: Data are available through the National Database for Autism Research (NDAR) accession number for MARBLES (2462).

Acknowledgements: N/A

**MMNP (Mumbai Maternal Nutrition Project)**

In the MMNP cohort, the children were born to mothers living in slums in the Bandra, Andheri and Khar districts of the city of Mumbai, India. These women had taken part in a food-based nutritional supplementation trial starting before the conception of the child. The pre-conceptional intervention was a daily snack, eaten in addition to normal diet, made from naturally micronutrient-rich local foods (green leafy vegetables, fruit and milk powder).^42^ Women were randomized to receive either 1 intervention or 1 control snack daily, 6 d per week, and intake was observed and recorded. Control snacks contained foods of low micronutrient content (e.g., potato, onion). On average, treatment snacks contained 10–23% of the WHO/FAO recommended Reference Nutrient Intakes for b-carotene, riboflavin, folate, vitamin B-12, calcium, and iron. Supplementation continued through pregnancy until delivery. The children were followed up at 5–7 y of age (N=1,255; “SARAS KIDS” study^43^), and data and whole blood samples for 689 children who were born to women who started supplementation at least 3 months prior to conception were used for the current analysis.

Peripheral blood samples from the 5–7 year-old children and their parents were collected and stored in EDTA-containing vacutainer tubes at −80°C. DNA was extracted using the QIAamp Midi DNA isolation kit (Qiagen) according to the manufacturer's protocol. Epigenome-wide DNA methylation profiling was performed using the Illumina MethylationEPIC BeadChip platform (Illumina). To reduce the potential for confounding by batch effects, samples were distributed on the plates following a balanced, randomized design, specified in advance. All exposure and covariate information were self-reported by participants. Intervention was included as a covariate in the models.

Normalization and QC steps:

Firstly, methylation predicted sex (as determined by the difference between median methylation of X and Y chromosome probes) was used to check sample labels and to detect and remove sex outliers, defined as those > 5 SDs from the mean. Next, the median methylated to unmethylated signal from the control probes was used to identify outlier samples and remove any that were > 3 SDs from the fitted regression line. Next, probe filtering was performed to remove probes with detection p-value > 0.01 and number of beads < 3 across more than 10% of samples. Finally, additional probe filtering was performed to exclude unreliable probes previously found to be multi-mapping or cross-hybridizing^21^, and probes mapping to X and Y chromosomes. For normalization, the standard meffil approach was used, which performs dye-bias and background correction using the noob method and an implementation of functional normalization. Batch as a covariate was used. Directly measured blood cell counts were used as covariates in the analysis.

Ethical approval: Ethics approval for the SARAS KIDS study was obtained from the Intersystem Biomedica Ethics Committee, Mumbai (ISBEC/NR-54/KM/JVJ/2013). Informed parental consent and the children’s assent were obtained.

MMNP (ISRCTN62811278) was approved by the ethics committees of BYL Nair and TN Medical College, Grant Medical College, and Sir JJ Group of Hospitals, Mumbai. Ethics approval for the follow-up of the children in Mumbai (“SARAS KIDS”) was obtained from the Intersystem Biomedica Ethics Committee, Mumbai on 31 May 2013 (serial no. ISBEC/NR-54/KM/JVJ/2013).  The EMPHASIS study is registered as ISRCTN14266771. Signed informed consent was obtained from parents and verbal assent from the children. Ethics approval for the molecular biology experiments conducted in CSIR-CCMB was obtained from the Institutional Animal Ethic Committee (Registration no. 20/GO/RBi/99/CPCSEA).

Funding: The SARAS KIDS study was funded by the Medical Research Council, UK (grant no: MR/M005186/1) and DNA methylation was measured in the EMPHASIS study^44^ (https://www.emphasisstudy.org/) which was jointly funded by MRC, Department for International Development, UK and the Department of Biotechnology (DBT), Ministry of Science and Technology, India, under the Newton Fund initiative (MRC Grant No.: MR/ N006208/1 and DBT Grant No.: BT/IN/DBT-MRC/DFID/24/GRC/2015–16). Core funds sanctioned by the Council of Scientific and Industrial Research (CSIR), Ministry of Science and Technology, Government of India, New Delhi, India were also utilized.

Conflicts of Interest: None

Data Availability: Requests to access the MMNP data should be submitted to Giriraj R Chandak, CSIR-Centre for Cellular and Molecular Biology (CSIR-CCMB), Hyderabad. INDIA (chandakgrc@ccmb.res.in).

Acknowledgements: We are grateful to the families who took part, the team of fieldworkers and nurses, research assistants and the data managers who carried out the study.

**The Norwegian Mother, Father and Child Cohort study (MoBa)**

The Norwegian Mother, Father and Child Cohort study (MoBa)^45^ is a cohort study with more than 110 000 children and their parents. Women were recruited during pregnancy between 1998 and 2008, and approximately 40% of those invited participated. Fathers were included from 2001. Participants are followed with questionnaires and by linkage to the Medical Birth Registry of Norway. Blood samples were collected from the parents during pregnancy, and from the umbilical cord at delivery.^46^ Participants in the current analysis represent 4 subsets of offspring samples, referred to here as: MoBa1, MoBa2, MoBa4, and MoBa8. Any overlapping samples between the four samples were accounted for and removed.

All the data sets described here where subjected to the same quality control (QC) and normalized using the same R package (RnBeads^47^), with the same parameters and the same normalization procedures. Briefly, all cross-hybridizing probes where removed, along with probes that had a common SNP on the CpG site, along with probes that had a high detection p-value (>0.01). Greedycut was used to remove outlier probes and samples, and the remaining samples where normalized using out-of-band normalization, with color correction. Empty wells and samples with technical issues were manually removed. The BMIQ algorithm was applied on the autosomal CpGs to normalized type I and type II chemistries on the Illumina BeadChip.

*MoBa1*

Participants for the MoBa1 samples were selected from the MoBa cohort, consisting of 1139 samples, typed on the Illumina 450k platform, out of which 19 were duplicates.^48^ After QC steps, including filtering of low-quality samples, samples with high levels of background noise, and samples with >10% missing, 1075 samples remained. In the v13 update of MoBa, 9 of these individuals had withdrawn their consent, leaving 1067 samples in the final MoBa1 methylation sample.

*MoBa2*

MoBa2 samples were selected from MoBa in a design to evaluate the association between maternal plasma folate during pregnancy and childhood asthma, and the role of DNA methylation in this relationship.^49^ This sample is significantly enriched for children who later developed asthma at age 3. The subset contained 817 samples, typed on the Illumina 450k platform, of which 89 where replicates. After QC and filtering, 688 samples remained. In the v13 update of MoBa, 4 of these mothers or children had withdrawn their consent, leaving 684 samples in the final subset.

*MoBa4*

The MoBa4 subset were designed to assess differences between children conceived with artificial reproductive techniques (ART), such as IVF and ICSI.^50^ 2000 triads, of which 1000 were randomly selected controls, where typed on the Illumina EPIC array. After QC, 1970 children remained in the sample, out of which 988 was in the random sample, which included 21(2.5%) conceived by ART. For the current meta-analysis, the ART group was excluded. In the v13 update of MoBa, 3 of these had withdrawn their consent, leaving 985 children in the final sample.

*MoBa8*

The MoBa8 samples were pulled randomly among all triads that had a blood sample and were genotyped. 1248 triads were selected for typing on the Illumina EPIC platform, giving a total of 3744 samples. After exclusion of technical samples, 1241 of the samples were children, of which 14 were replicates. After QC, 1215 samples remained, and in the v13 update of MoBa, none of the participant had withdrawn their consent, leaving 1215 children in the final sample.

Ethical Approval: This study was approved by the Regional Committees for Medical and Health Research Ethics of South East Norway (REK #2017/1362). All participants provided written informed consent. The establishment of MoBa and initial data collection were based on a license from the Norwegian Data Protection Agency and an approval from the Regional Committees for Medical and Health Research Ethics. The MoBa cohort is now regulated by the Norwegian Health Registry Act.

Funding: This work was funded by the Research Council of Norway through its Centres of Excellence funding scheme, project number 262700. Methylation measurements in MoBa1 and MoBa2 were supported by the Intramural Research Program of the NIH, National Institute of Environmental Health Sciences (NIH/NIEHS contract no N01-ES-75558 and ZO1 ES49019).

Conflicts of Interest: None

Data Availability: The data that support the findings of this study are available from the Norwegian Institute of Public Health (NIPH), but restrictions apply regarding the availability of these data, which were originally used under specific approvals for the current study and are therefore not publicly available. The individual level data are available under restricted access due to regulations and access can only be given after approval by the Norwegian Ethical committees under the provision that the applications are consistent with the consent provided. Access can be obtained by application to the Norwegian Institute of Public Health using a form available on the English language portion of its website at https://www.fhi.no/en/studies/moba/. Specific questions regarding access for data in this study can be directed to Siri.Haberg@fhi.no.

Acknowledgements: The Norwegian Mother, Father, and Child Cohort Study is supported by the Norwegian Ministry of Health and Care Services and the Ministry of Education and Research. We are grateful to all the participating families who take part in this ongoing cohort study.

**NorthPop**

Since 2016, all pregnant women in Västerbotten are invited to participate in the NorthPop Birth Cohort Study together with their partner and child (current pregnancy). Please see NorthPop's homepage for full information on data collection (<https://www.northpop.se/en/home-2/>). Families are followed longitudinally until the child is 7 years old. The data collection includes web-based questionnaires and biological samples. During pregnancy, questionnaires are answered by the mother and the partner. A series of questionnaires are administered when the child is 4 months, 9 months, 18 months, 2 years, 3 years, and 7 years old. Umbilical cord blood is collected in EDTA vacutainer tubes and separated into plasma, buffy coat and erythrocyte fractions using centrifugation. In the current study, DNA was extracted from 200 µL buffy coat samples using the FlexiGene DNA kit (Qiagen), according to the standard protocol, and then eluted and stored in Tris-EDTA buffer. Concentration and purity were determined using NanoDrop (Thermo Fisher Scientific). All samples were handled and stored until analysis at Biobanken norr, Umeå, Sweden. Mothers and their partners were solicited for information. Hence, covariate information was based on self-report. We included 722 newborns with DNA methylation data of whom, 679 were from primipara singleton pregnancies whereas the additional 43 children were from multiple-birth (twin or triplet) pregnancies which included women of different parity (22 primipara 21 multipara). For paternal age models, 706 remained after excluding for those where partners were not male or gender was unknown. It is likely only very few samples where the male partner was not the biological father.

Methylation profiling was performed with the Infinium MethylationEPIC BeadChip and the results were analyzed with GenomeStudio 2011.1 from Illumina Inc. All samples included for analysis in this project passed Illumina’s ’Probe call rate’ > 0.98 limit. The ENmix package was used to pre-process the methylation data. Before preprocessing, SNP-related probes, probes with call rate P-value<0.01 and probes with missing in more than 20% of samples were removed. Background correction of methylation signal intensities was made with the ENmix-algorithm. We applied inter-array normalization with the quantile method and probe-type bias adjustment using the RCP (Regression on Correlated Probes) method. Finally, cell counts were estimated using a cord blood reference^14^ in minfi and FlowSorted.Blood. EPIC packages. Adjustment for batch was done by including plate identification.

Ethical Approval: The NorthPop study was approved by the Research Ethics Committee in Umeå, Sweden, 2014/224-31. Written informed consent was obtained from both parents.

Funding: The NorthPop infrastructure receives funding from Västerbotten County Council and Umeå University (MD and CEW). DNA extraction, methylation profiling and data analyses were funded by grants from the Swedish Asthma and allergy foundation grant number: F2018-0027 (SH), the Swedish Research Council grant number 2019-01187 (SH), the Swedish Heart-Lung Foundation grant number 2020-0473 (SH) and FORMAS, grant number 2021-01098 (SH). The funding bodies had no role in study design, data collection and analysis nor in the preparation of the manuscript.

Conflicts of Interest: None

Data Availability: Data described in the manuscript, will be made available upon reasonable request pending valid ethical approval as well as approval by the NorthPop steering committee.

Acknowledgements: We acknowledge all participating families in the NorthPop study; the NorthPop project team for recruitment, follow-up, and blood samplings of study participants; the NorthPop coordinator Richard Lundberg at the Department of Clinical Sciences, Umeå University and the personnel at Biobanken Norr,, Västerbotten county council. Methylation profiling was performed by the SNP&SEQ Technology Platform in Uppsala (www.genotyping.se). The facility is part of the National Genomics Infrastructure (NGI) Sweden and Science for Life Laboratory. The SNP&SEQ Platform is also supported by the Swedish Research Council and the Knut and Alice Wallenberg Foundation.

**PMMST (Peri-conceptional Multiple Micronutrient Supplementation Trial)**

PMMST (ISRCTN13687662) was a periconceptional micronutrient supplementation trial conducted in rural Gambia in Sub-Saharan West Africa to investigate the effect of micronutrient supplementation on placental function.^51^ Children born to PMMST mothers were followed up at 7-9 years as a part of the EMPHASIS study to investigate (epi)genetic links to maternal exposures and childhood health.^44^ There were 376 singleton live births between 2007 and 2008 and the current study includes 293 children with DNA available.

Data collection methods

Peripheral blood samples from individuals were collected at the time of 7 – 9 years follow-up and DNA was isolated using blood DNA isolation kits (QIAamp blood DNA isolation kit, Qiagen). Illumina InfiniumEPIC array (Illumina, USA) was used to measure genome-wide methylation covering 850K CpG loci. Parental phenotype data was collected at the time of recruitment prior to pregnancy and at the time of delivery. Maternal and paternal age came from self-report. Genotype PCs 1 to 4 generated from child’s genotype data were included as covariates to proxy ancestry / tribal differences. The Houseman method cell type estimation was used to derive estimate CD8T, CD4T, NK, Bcell, Mono, and Gran cell counts. Smoking is not practiced in this population hence no smoking covariates were included in any of the models. Parental education levels and parity information were not available.

Normalization and QC steps

First pass QC of the EPIC array DNAm data was carried out in GenomeStudio to assess the success of the experimental steps. Next, the raw .idat files were imported to R statistical environment and the data was normalized using the meffil R package following the Functional Normalisation default parameters.^52^ X and Y probes and known cross-reactive probes^21^ were removed. Post QC, there were 289 children included in the current analysis.

Ethical approval: This trial was approved by the Scientific Coordinating Committee of MRC Laboratories, The Gambia, and by the MRC/Gambian Government Ethics Committee (L2005.111v2 SCC 1000).

Funding: PMMST was supported by Medical Research Council (MRC) grants U1232661351, U105960371 and MC-A760-5QX00 and by the UK Government Department for International Development (DFID) under the MRC/DFID Concordat. The EMPHASIS study is jointly funded by MRC, DFID, and the Department of Biotechnology (DBT), Ministry of Science and Technology, India under the Newton Fund initiative (MRC grant MR/N006208/1. Analysts were additionally supported by MRC grant MR/T032863/1.

Conflicts of Interest: None

Data Availability: Requests to access the Gambian data should be submitted to Prof Andrew Prentice (andrew.prentice@lsthm.ac.uk) in the first instance. An application would then need to be made to MRC Unit The Gambia’s Scientific Coordinating Committee and the Joint MRC/Gambia Government Ethics Committee.

Acknowledgements: We would like to thank all the study participants, lab staff and the field team in West Kiang, The Gambia for their contributions to the original study.

**POSEIDON (Pre-, Peri-, and POstnatal Stress: Epigenetic Impact on DepressiON)**

Four hundred and ten women agreed to participate in a longitudinal study on perceived stress and child development and health. Recruitment started in 2010. So far, four waves were conducted: during the third trimester of pregnancy (T1), at childbirth (T2), 6 months postpartum (T3) and at 45 months postpartum (T4). All women who participated lived in the Rhine-Neckar-Region in Germany. The total sample consisted of 410 mother-newborn dyads. 277 newborns and 277 mothers were Caucasian. Maternal exclusion criteria for the present analyses were: (i) a history of hepatitis B, hepatitis C, or HIV-infection; (ii) any current or previous diagnosis of schizophrenia or any substance dependency other than nicotine,(iii) any current psychiatric disorder requiring inpatient treatment. Exclusion criteria in the offspring were: birth weight <1.500 g; gestational age at delivery <32 weeks; multiple birth; and the presence of a congenital disease, malformation, deformation, or chromosomal abnormality; and (iv) non-caucasian ancestry.^53^ Whole cord blood was collected immediately after birth from n=331 newborn singletons. DNA methylation was measured using the Illumina Infinium HumanMethylation450K Beadchip.

Intensity data were extracted from raw data (idat) files using a verified pipeline.^31^ Samples were excluded due to: insufficient DNA quality, insufficient bisulfite conversion; or failure in detection (detection P-value > 0.01 at more than 1% of positions), or sex-mismatch between phenotype and methylation data. Quality control steps included: detection p-value threshold (positions/sites) > 0.01, call rate 95% (samples), and exclusion of sex chromosomes, and quantile normalization. After quality control processing, 473,864 probes remained for analysis. Cell count estimation was performed using a cord blood reference.^14^

Information on the exposure and covariates: In Germany, there is a central documentation booklet "Mutterpass" every mother needs to carry. All relevant information determined by physicians and midwifes are documented in the Mutterpass. Information on birth measures of the child such as sex, birth weight, and gestational age were derived from the Mutterpass. Information on age (both maternal and paternal) were obtained by self-reports of the participants. Also, smoking behavior during pregnancy and previous pregnancies of the mother (parity) were assessed by self-reports. Maternal BMI was taken from the Mutterpass and paternal BMI was derived from self-reported weight and height. Maternal and paternal education were measured based on self-reports and the ISCED 2011 levels were used to transform the highest parental education attainment in our sample. Afterwards the ISCED levels were transformed into US years-of-schooling. As only one ethnicity group (White/Caucasian) was present, genotype PCs were used to correct for residual ancestry of mothers and fathers within this group. The first 4 genotype PCs were used (genotype QC based on PLINK v1.9).

Ethical Approval: The study protocol was approved by the Ethics Committee of the Medical Faculty Mannheim of the University of Heidelberg. The study was conducted in accordance with the Declaration of Helsinki.

Funding: This work was supported by the German Research Foundation [DFG; grant FOR2107; RI908/11-2 and WI3429/3-2], the German Federal Ministry of Education and Research (BMBF) through the Integrated Network IntegraMent, under the auspices of the e:Med Programme [01ZX1314G; 01ZX1614G] through grants 01EE1406C, 01EE1409C and through ERA-NET NEURON, “SynSchiz - Linking synaptic dysfunction to disease mechanisms in schizophrenia - a multilevel investigation“ [01EW1810], through ERA-NET NEURON “Impact of Early life MetaBolic and psychosocial strEss on susceptibility to mental Disorders; from converging epigenetic signatures to novel targets for therapeutic intervention” [01EW1904] and by a grant of the Dietmar-Hopp Foundation.

Conflicts of Interest: The authors declare no conflict of interest.

Data Availability: Data from the POSEIDON cohort are not publicly available due legal restrictions but are available upon reasonable request.

Acknowledgements: We thank all parents and children for taking part in this study.

**Upstate KIDS (Upstate New York Infant Development Screening) Study**

Upstate KIDS is a longitudinal birth cohort that originally enrolled 5,034 mothers and 6,171 infants born between 2008-2010 in New York State (excluding New York City).^54^ The cohort oversampled singletons conceived by infertility treatment and multiples. Follow-up of cohort participants continued until 2019.^55^ A subset of 733 singletons and twins (one randomly selected twin from each pair in the same family) with newborn DNA methylation measured and 146 with childhood DNA methylation measured were included in the current analysis, after exclusion of participants who declined consent of secondary uses of methylation information. A subgroup of children attended clinic visits when they were 8-10 years old. Blood was processed into buffy coat samples and frozen in -80^o^C storage until DNA extraction of samples. Parental age, parity, infant sex, gestational age, birthweight, and race/ethnicity were extracted from vital records. Parental information on education, weight and height for deriving body mass index, and smoking status were reported at baseline by mothers at approximately 4 months postpartum.

Newborn dried blood spots (DBS) were retrieved from the New York State’s Newborn Screening Program.^56^ DNA was extracted from DBS punches using the GenSolve DNA recovery kit (GenTegra, Pleasanton, CA) followed by purification with QIAmp DNA kits (#51104, QIAGEN, Valencia, CA).^57^ DNA from DBS and from buffy coat samples then underwent bisulfite conversion with standardized kits (Zymo EZ DNA Methylation kit; Zymo, Irvine, CA). Methylation was measured using the Infinium MethylationEPIC 850K BeadChip microarray (Illumina, San Diego, CA) and was processed using the minfi package in R.^28^ Quantile normalization, background, and dye-bias adjustments of CpG probes was applied.^55^ After QC steps, 837,234 CpG probes remained for newborn analysis and 833,253 CpG probes remained for childhood analysis. Cell count proportions in DBS were estimated from a cord blood reference^58^ while cell count from circulating samples was estimated by Houseman method.^59^ Plate number was used to adjust for batch. Infant’s DNAm-derived ancestry was inferred using GLINT.^60^ Fertility treatment was included as a covariate in the model.

Ethical approval: The New York State Department of Health and the University at Albany (State University of New York) institutional review boards (IRB) approved the study. Parents provided written informed consent for the retrieval of remaining newborn dried blood spots (DBS) from New York State’s Newborn Screening program when the infants were 8 months old, as well as additional genetic analysis consent in 2016-2017.^56^ Parental consent and child assent was provided at clinic visits when children were 9 years old prior to study exam and blood draw.

Funding: Supported by the Intramural Research Program of the *Eunice Kennedy Shriver* National Institute of Child Health and Human Development (NICHD; contracts #HHSN275201200005C, #HHSN267200700019C, #HHSN275201400013C, #HHSN275201300026I/27500004, #HHSN275201300023I/27500017).

Conflicts of interest: None

Data availability: The data that support the findings of this study are available on request from the corresponding author [EY]. The data are not on a public database due to New York State restrictions (i.e., releasing information that could compromise participant privacy/consent).

Acknowledgements: We thank the Upstate KIDS participants and staff for their important contributions. This work utilized the computational resources of the NIH HPC Biowulf cluster (http://hpc.nih.gov).

Supplemental background

*SHANK1*

*The family of SHANK (SH3 and multiple ankyrin repeat containing protein) proteins serves to regulate synaptic transmission in neuronal tissue, particularly acting as a principal scaffold at postsynaptic sites.^61^ All SHANK mutations including SHANK1 gene variants have been associated with a range of neurodevelopmental disorders including autism. SHANK1 is almost exclusively expressed in the brain, particularly in the hippocampus, amygdala, and cortex.^61^ Less is known regarding its methylation and its impact on disorders. Differential methylation of a region of CLEC11A (next to SHANK1) was identified in whole blood of 75 siblings discordant for autism, independent of genotype.^62^ With regard to developmental programming, altered expression of SHANK1 in mouse offspring brain tissue followed after exposure to maternal high fat diet, which correlated with autistic traits.^63,64^ In a look-up of the EWAS catalog^65^, two studies found methylation associations with age and sex^66,67^, one study had previously identified differences in methylation with atopy^68^ and one with renal cell carcinoma.^69^*

*C2orf81*

*Two CpGs in C2orf81 (cg03292743,* *cg24129222) were identified with maternal age as a DMR in newborns but methylation levels at these two sites were not associated with maternal age in childhood blood samples. C2orf81 (chromosome 2 open reading frame 81) is particularly expressed in reproductive tissues according to the Human Protein Atlas (i.e., testis and fallopian tube). Other than age and sex, the EWAS catalog identified associations with varied outcomes in the literature including rheumatoid arthritis, ulcerative colitis, HIV infection, and renal cell carcinoma.^65^*

*BOLA2B/YPEL3*

*Three CpGs identified (cg26709300, cg16348385, cg27106909) as a DMR with increasing maternal age and decreasing newborn DNA methylation were in BOLA2B (intron)/YPEL3 (exon). BOLA2B (bolA homolog 2) is involved in iron maturation and related to multiple cancers.^70^ YPEL3 (yippee-like 3) is ubiquitously expressed and a target for tumor suppressor p53, which helps regulate cell senescence.^71^ In a look-up in the EWAS catalog^65^, levels of 6 proteins were correlated with methylation in the Generation Scotland study.^72^ These included GP1BA, IGF1R, PRSS57, VWA2, EPO, and IL12B. The study used the EPIC microarray to measure DNA methylation among 774 older adults who also had proteomic data by SOMAscan V.4. The HELIX reference of childhood blood samples had identified ITGAL. However, all three CpGs were also previously identified to be inversely associated with gestational age in three separate studies^73-75^ and positively with birthweight in PACE.^76^ Thus, these CpG associations, unlike MTNR1B, may be driven by advanced maternal age increasing risk of preterm delivery and/or low birth weight.*

1 Boyd, A. *et al.* Cohort Profile: the 'children of the 90s'--the index offspring of the Avon Longitudinal Study of Parents and Children. *Int J Epidemiol* **42**, 111-127 (2013). <https://doi.org/10.1093/ije/dys064>

2 Fraser, A. *et al.* Cohort Profile: the Avon Longitudinal Study of Parents and Children: ALSPAC mothers cohort. *Int J Epidemiol* **42**, 97-110 (2013). <https://doi.org/10.1093/ije/dys066>

3 Relton, C. L. *et al.* Data Resource Profile: Accessible Resource for Integrated Epigenomic Studies (ARIES). *Int J Epidemiol* **44**, 1181-1190 (2015). <https://doi.org/10.1093/ije/dyv072>

4 Min, J. L., Hemani, G., Davey Smith, G., Relton, C. & Suderman, M. Meffil: efficient normalization and analysis of very large DNA methylation datasets. *Bioinformatics* **34**, 3983-3989 (2018). <https://doi.org/10.1093/bioinformatics/bty476>

5 Bristol, U. o. *Avon Longitudinal Study of Parents and Children: Questionnaires*, <<https://www.bristol.ac.uk/alspac/researchers/our-data/questionnaires/>> (

6 Gervin, K. *et al.* Cell type specific DNA methylation in cord blood: A 450K-reference data set and cell count-based validation of estimated cell type composition. *Epigenetics* **11**, 690-698 (2016). <https://doi.org/10.1080/15592294.2016.1214782>

7 Eskenazi, B. *et al.* CHAMACOS, A Longitudinal Birth Cohort Study: Lessons from the Fields. *Journal of Children's Health* **1**, 3-27 (2003). <https://doi.org/10.3109/713610244>

8 Holland, N. *et al.* Paraoxonase polymorphisms, haplotypes, and enzyme activity in Latino mothers and newborns. *Environ Health Perspect* **114**, 985-991 (2006). <https://doi.org/10.1289/ehp.8540>

9 Bibikova, M. *et al.* High density DNA methylation array with single CpG site resolution. *Genomics* **98**, 288-295 (2011). <https://doi.org/10.1016/j.ygeno.2011.07.007>

10 Sandoval, J. *et al.* Validation of a DNA methylation microarray for 450,000 CpG sites in the human genome. *Epigenetics* **6**, 692-702 (2011). <https://doi.org/10.4161/epi.6.6.16196>

11 Yousefi, P. *et al.* Sex differences in DNA methylation assessed by 450 K BeadChip in newborns. *BMC Genomics* **16**, 911 (2015). <https://doi.org/10.1186/s12864-015-2034-y>

12 Aryee, M. J. *et al.* Minfi: a flexible and comprehensive Bioconductor package for the analysis of Infinium DNA methylation microarrays. *Bioinformatics* **30**, 1363-1369 (2014). <https://doi.org/10.1093/bioinformatics/btu049>

13 Solomon, O. *et al.* Comparison of DNA methylation measured by Illumina 450K and EPIC BeadChips in blood of newborns and 14-year-old children. *Epigenetics* **13**, 655-664 (2018). <https://doi.org/10.1080/15592294.2018.1497386>

14 Gervin, K. *et al.* Systematic evaluation and validation of reference and library selection methods for deconvolution of cord blood DNA methylation data. *Clin Epigenetics* **11**, 125 (2019). <https://doi.org/10.1186/s13148-019-0717-y>

15 McConnell, R. *et al.* Traffic, susceptibility, and childhood asthma. *Environ Health Perspect* **114**, 766-772 (2006). <https://doi.org/10.1289/ehp.8594>

16 Noushmehr, H. *et al.* Identification of a CpG island methylator phenotype that defines a distinct subgroup of glioma. *Cancer Cell* **17**, 510-522 (2010). <https://doi.org/10.1016/j.ccr.2010.03.017>

17 Triche, T. J., Jr., Weisenberger, D. J., Van Den Berg, D., Laird, P. W. & Siegmund, K. D. Low-level processing of Illumina Infinium DNA Methylation BeadArrays. *Nucleic Acids Res* **41**, e90 (2013). <https://doi.org/10.1093/nar/gkt090>

18 Touleimat, N. & Tost, J. Complete pipeline for Infinium((R)) Human Methylation 450K BeadChip data processing using subset quantile normalization for accurate DNA methylation estimation. *Epigenomics* **4**, 325-341 (2012). <https://doi.org/10.2217/epi.12.21>

19 Morin, A. M. *et al.* Maternal blood contamination of collected cord blood can be identified using DNA methylation at three CpGs. *Clin Epigenetics* **9**, 75 (2017). <https://doi.org/10.1186/s13148-017-0370-2>

20 Fortin, J. P. *et al.* Functional normalization of 450k methylation array data improves replication in large cancer studies. *Genome Biol* **15**, 503 (2014). <https://doi.org/10.1186/s13059-014-0503-2>

21 Pidsley, R. *et al.* Critical evaluation of the Illumina MethylationEPIC BeadChip microarray for whole-genome DNA methylation profiling. *Genome Biol* **17**, 208 (2016). <https://doi.org/10.1186/s13059-016-1066-1>

22 Price, M. E. *et al.* Additional annotation enhances potential for biologically-relevant analysis of the Illumina Infinium HumanMethylation450 BeadChip array. *Epigenetics Chromatin* **6**, 4 (2013). <https://doi.org/10.1186/1756-8935-6-4>

23 Leek, J. T., Johnson, W. E., Parker, H. S., Jaffe, A. E. & Storey, J. D. The sva package for removing batch effects and other unwanted variation in high-throughput experiments. *Bioinformatics* **28**, 882-883 (2012). <https://doi.org/10.1093/bioinformatics/bts034>

24 Koestler, D. C. *et al.* Improving cell mixture deconvolution by identifying optimal DNA methylation libraries (IDOL). *BMC Bioinformatics* **17**, 120 (2016). <https://doi.org/10.1186/s12859-016-0943-7>

25 Schisterman, E. F. *et al.* Preconception low-dose aspirin and pregnancy outcomes: results from the EAGeR randomised trial. *Lancet* **384**, 29-36 (2014). <https://doi.org/10.1016/s0140-6736(14)60157-4>

26 Schisterman, E. F. *et al.* A randomised trial to evaluate the effects of low-dose aspirin in gestation and reproduction: design and baseline characteristics. *Paediatr Perinat Epidemiol* **27**, 598-609 (2013). <https://doi.org/10.1111/ppe.12088>

27 Yeung, E. H. *et al.* Measured maternal prepregnancy anthropometry and newborn DNA methylation. *Epigenomics* **11**, 187-198 (2019). <https://doi.org/10.2217/epi-2018-0099>

28 Aryee, M. J. *et al.* Minfi: a flexible and comprehensive Bioconductor package for the analysis of Infinium DNA methylation microarrays. *Bioinformatics* **30**, 1363-1369 (2014). <https://doi.org/10.1093/bioinformatics/btu049>

29 Newschaffer, C. J. *et al.* Infant siblings and the investigation of autism risk factors. *J Neurodev Disord* **4**, 7 (2012). <https://doi.org/10.1186/1866-1955-4-7>

30 Kooijman, M. N. *et al.* The Generation R Study: design and cohort update 2017. *Eur J Epidemiol* **31**, 1243-1264 (2016). <https://doi.org/10.1007/s10654-016-0224-9>

31 Lehne, B. *et al.* A coherent approach for analysis of the Illumina HumanMethylation450 BeadChip improves data quality and performance in epigenome-wide association studies. *Genome Biol* **16**, 37 (2015). <https://doi.org/10.1186/s13059-015-0600-x>

32 Guillemette, L. *et al.* Genetics of Glucose regulation in Gestation and Growth (Gen3G): a prospective prebirth cohort of mother-child pairs in Sherbrooke, Canada. *BMJ Open* **6**, e010031 (2016). <https://doi.org/10.1136/bmjopen-2015-010031>

33 Juvinao-Quintero, D. L. *et al.* Epigenome-wide association study of maternal hemoglobin A1c in pregnancy and cord blood DNA methylation. *Epigenomics* **13**, 203-218 (2021). <https://doi.org/10.2217/epi-2020-0279>

34 Niu, L., Xu, Z. & Taylor, J. A. RCP: a novel probe design bias correction method for Illumina Methylation BeadChip. *Bioinformatics* **32**, 2659-2663 (2016). <https://doi.org/10.1093/bioinformatics/btw285>

35 Maitre, L. *et al.* Human Early Life Exposome (HELIX) study: a European population-based exposome cohort. *BMJ Open* **8**, e021311 (2018). <https://doi.org/10.1136/bmjopen-2017-021311>

36 Vrijheid, M. *et al.* The human early-life exposome (HELIX): project rationale and design. *Environ Health Perspect* **122**, 535-544 (2014). <https://doi.org/10.1289/ehp.1307204>

37 van Iterson, M. *et al.* MethylAid: visual and interactive quality control of large Illumina 450k datasets. *Bioinformatics* **30**, 3435-3437 (2014). <https://doi.org/10.1093/bioinformatics/btu566>

38 Fortin, J. P., Fertig, E. & Hansen, K. shinyMethyl: interactive quality control of Illumina 450k DNA methylation arrays in R. *F1000Res* **3**, 175 (2014). <https://doi.org/10.12688/f1000research.4680.2>

39 Johnson, W. E., Li, C. & Rabinovic, A. Adjusting batch effects in microarray expression data using empirical Bayes methods. *Biostatistics* **8**, 118-127 (2007). <https://doi.org/10.1093/biostatistics/kxj037>

40 Salas, L. A. *et al.* Enhanced cell deconvolution of peripheral blood using DNA methylation for high-resolution immune profiling. *Nat Commun* **13**, 761 (2022). <https://doi.org/10.1038/s41467-021-27864-7>

41 Hertz-Picciotto, I. *et al.* A Prospective Study of Environmental Exposures and Early Biomarkers in Autism Spectrum Disorder: Design, Protocols, and Preliminary Data from the MARBLES Study. *Environ Health Perspect* **126**, 117004 (2018). <https://doi.org/10.1289/EHP535>

42 Potdar, R. D. *et al.* Improving women's diet quality preconceptionally and during gestation: effects on birth weight and prevalence of low birth weight--a randomized controlled efficacy trial in India (Mumbai Maternal Nutrition Project). *Am J Clin Nutr* **100**, 1257-1268 (2014). <https://doi.org/10.3945/ajcn.114.084921>

43 Sahariah, S. A. *et al.* Body Composition and Cardiometabolic Risk Markers in Children of Women who Took Part in a Randomized Controlled Trial of a Preconceptional Nutritional Intervention in Mumbai, India. *J Nutr* **152**, 1070-1081 (2022). <https://doi.org/10.1093/jn/nxab443>

44 Chandak, G. R. *et al.* Protocol for the EMPHASIS study; epigenetic mechanisms linking maternal pre-conceptional nutrition and children's health in India and Sub-Saharan Africa. *BMC Nutr* **3** (2017). <https://doi.org/10.1186/s40795-017-0200-0>

45 Magnus, P. *et al.* Cohort Profile Update: The Norwegian Mother and Child Cohort Study (MoBa). *Int J Epidemiol* **45**, 382-388 (2016). <https://doi.org/10.1093/ije/dyw029>

46 Ronningen, K. S. *et al.* The biobank of the Norwegian Mother and Child Cohort Study: a resource for the next 100 years. *Eur J Epidemiol* **21**, 619-625 (2006). <https://doi.org/10.1007/s10654-006-9041-x>

47 Muller, F. *et al.* RnBeads 2.0: comprehensive analysis of DNA methylation data. *Genome Biol* **20**, 55 (2019). <https://doi.org/10.1186/s13059-019-1664-9>

48 Joubert, B. R. *et al.* 450K epigenome-wide scan identifies differential DNA methylation in newborns related to maternal smoking during pregnancy. *Environ Health Perspect* **120**, 1425-1431 (2012). <https://doi.org/10.1289/ehp.1205412>

49 Joubert, B. R. *et al.* DNA Methylation in Newborns and Maternal Smoking in Pregnancy: Genome-wide Consortium Meta-analysis. *Am J Hum Genet* **98**, 680-696 (2016). <https://doi.org/10.1016/j.ajhg.2016.02.019>

50 Haberg, S. E. *et al.* DNA methylation in newborns conceived by assisted reproductive technology. *Nat Commun* **13**, 1896 (2022). <https://doi.org/10.1038/s41467-022-29540-w>

51 Owens, S. *et al.* Periconceptional multiple-micronutrient supplementation and placental function in rural Gambian women: a double-blind, randomized, placebo-controlled trial. *Am J Clin Nutr* **102**, 1450-1459 (2015). <https://doi.org/10.3945/ajcn.113.072413>

52 Saffari, A. *et al.* Effect of maternal preconceptional and pregnancy micronutrient interventions on children's DNA methylation: Findings from the EMPHASIS study. *Am J Clin Nutr* **112**, 1099-1113 (2020). <https://doi.org/10.1093/ajcn/nqaa193>

53 Witt, S. H. *et al.* Impact on birth weight of maternal smoking throughout pregnancy mediated by DNA methylation. *BMC Genomics* **19**, 290 (2018). <https://doi.org/10.1186/s12864-018-4652-7>

54 Buck Louis, G. M. *et al.* Methodology for Establishing a Population-Based Birth Cohort Focusing on Couple Fertility and Children's Development, the Upstate KIDS Study. *Paediatric and Perinatal Epidemiology* **28**, 191-202 (2014). <https://doi.org/https://doi.org/10.1111/ppe.12121>

55 Yeung, E. H. *et al.* Conception by fertility treatment and offspring deoxyribonucleic acid methylation. *Fertil Steril* **116**, 493-504 (2021). <https://doi.org/10.1016/j.fertnstert.2021.03.011>

56 Yeung, E. H. *et al.* Eliciting parental support for the use of newborn blood spots for pediatric research. *BMC Medical Research Methodology* **16** (2016). <https://doi.org/10.1186/s12874-016-0120-8>

57 Andersen, N. J. *et al.* Detection of immunoglobulin isotypes from dried blood spots. *Journal of Immunological Methods* **404**, 24-32 (2014). <https://doi.org/https://doi.org/10.1016/j.jim.2013.12.001>

58 Gervin, K. *et al.* Systematic evaluation and validation of reference and library selection methods for deconvolution of cord blood DNA methylation data. *Clin Epigenetics* **11**, 125 (2019). <https://doi.org/10.1186/s13148-019-0717-y>

59 Houseman, E. A., Kelsey, K. T., Wiencke, J. K. & Marsit, C. J. Cell-composition effects in the analysis of DNA methylation array data: a mathematical perspective. *BMC Bioinformatics* **16**, 95 (2015). <https://doi.org/10.1186/s12859-015-0527-y>

60 Rahmani, E. *et al.* GLINT: a user-friendly toolset for the analysis of high-throughput DNA-methylation array data. *Bioinformatics* **33**, 1870-1872 (2017). <https://doi.org/10.1093/bioinformatics/btx059>

61 Sheng, M. & Kim, E. The Shank family of scaffold proteins. *J Cell Sci* **113 ( Pt 11)**, 1851-1856 (2000). <https://doi.org/10.1242/jcs.113.11.1851>

62 Perini, S., Filosi, M., Italian Autism, N. & Domenici, E. Candidate biomarkers from the integration of methylation and gene expression in discordant autistic sibling pairs. *Transl Psychiatry* **13**, 109 (2023). <https://doi.org/10.1038/s41398-023-02407-4>

63 Gawlinska, K. *et al.* A Maternal High-Fat Diet during Early Development Provokes Molecular Changes Related to Autism Spectrum Disorder in the Rat Offspring Brain. *Nutrients* **13** (2021). <https://doi.org/10.3390/nu13093212>

64 Gawlinska, K., Gawlinski, D., Kowal-Wisniewska, E., Jarmuz-Szymczak, M. & Filip, M. Alteration of the Early Development Environment by Maternal Diet and the Occurrence of Autistic-like Phenotypes in Rat Offspring. *Int J Mol Sci* **22** (2021). <https://doi.org/10.3390/ijms22189662>

65 Battram, T. *et al.* The EWAS Catalog: a database of epigenome-wide association studies. *Wellcome Open Res* **7**, 41 (2022). <https://doi.org/10.12688/wellcomeopenres.17598.2>

66 Mulder, R. H. *et al.* Epigenome-wide change and variation in DNA methylation in childhood: trajectories from birth to late adolescence. *Hum Mol Genet* **30**, 119-134 (2021). <https://doi.org/10.1093/hmg/ddaa280>

67 Singmann, P. *et al.* Characterization of whole-genome autosomal differences of DNA methylation between men and women. *Epigenetics Chromatin* **8**, 43 (2015). <https://doi.org/10.1186/s13072-015-0035-3>

68 Kim, K. W. *et al.* Integrated genetic and epigenetic analyses uncover MSI2 association with allergic inflammation. *J Allergy Clin Immunol* **147**, 1453-1463 (2021). <https://doi.org/10.1016/j.jaci.2020.06.040>

69 Wozniak, M. B. *et al.* Integrative genome-wide gene expression profiling of clear cell renal cell carcinoma in Czech Republic and in the United States. *PLoS One* **8**, e57886 (2013). <https://doi.org/10.1371/journal.pone.0057886>

70 Liang, M. *et al.* Integrative analysis of the role of BOLA2B in human pan-cancer. *Front Genet* **14**, 1077126 (2023). <https://doi.org/10.3389/fgene.2023.1077126>

71 Kwon, Y. *et al.* YPEL3 expression induces cellular senescence via the Hippo signaling pathway in human breast cancer cells. *Toxicol Res* **39**, 711-719 (2023). <https://doi.org/10.1007/s43188-023-00208-x>

72 Gadd, D. A. *et al.* Integrated methylome and phenome study of the circulating proteome reveals markers pertinent to brain health. *Nat Commun* **13**, 4670 (2022). <https://doi.org/10.1038/s41467-022-32319-8>

73 Hannon, E. *et al.* Variable DNA methylation in neonates mediates the association between prenatal smoking and birth weight. *Philos Trans R Soc Lond B Biol Sci* **374**, 20180120 (2019). <https://doi.org/10.1098/rstb.2018.0120>

74 Spiers, H. *et al.* Methylomic trajectories across human fetal brain development. *Genome Res* **25**, 338-352 (2015). <https://doi.org/10.1101/gr.180273.114>

75 Kashima, K. *et al.* Identification of epigenetic memory candidates associated with gestational age at birth through analysis of methylome and transcriptional data. *Sci Rep* **11**, 3381 (2021). <https://doi.org/10.1038/s41598-021-83016-3>

76 Kupers, L. K. *et al.* Meta-analysis of epigenome-wide association studies in neonates reveals widespread differential DNA methylation associated with birthweight. *Nat Commun* **10**, 1893 (2019). <https://doi.org/10.1038/s41467-019-09671-3>
